# Astrocyte Mitochondrial UCP4 Reprograms Neuronal Network Oscillations via GDNF-Dependent K^+^-Ca^2+^ Signaling in Alzheimer’s Disease Mice

**DOI:** 10.3390/cells15070597

**Published:** 2026-03-27

**Authors:** Aisylu Gaifullina, Chaima Belhi, Leonardo Restivo, Jean-Yves Chatton

**Affiliations:** 1 Department of Fundamental Neurosciences, University of Lausanne, CH-1005 Lausanne, Switzerland; aisylu.gaifullina@unil.ch (A.G.); chaima.belhi@unil.ch (C.B.); leonardo.restivo@unil.ch (L.R.); 2 Cellular Imaging Facility, University of Lausanne, CH-1005 Lausanne, Switzerland

**Keywords:** mitochondria, astrocyte, sharp wave ripples, uncoupling protein, Alzheimer’s disease, GDNF

## Abstract

**Highlights:**

**What are the main findings?**

- Astrocytic UCP4 overexpression normalizes neuronal excitability and hippocampal/subicular microcircuits via modulation of ionic membrane currents in 3xTG Alzheimer’s disease mouse model.
- Astrocyte UCP4 upregulation increases GDNF levels, a neurotrophic factor known to support neuronal function.

**What is the implication of the main finding?**

- Astrocyte UCP4 promoting GDNF signaling represents a promising strategy for enhancing neuronal resilience in neurodegeneration.
- Targeting astrocyte mitochondria shows therapeutic potential even in symptomatic Alzheimer’s disease mice.

**Abstract:**

Neuron-targeted therapies for Alzheimer’s disease (AD) have shown limited efficacy, highlighting the need to explore glial-based mechanisms of neuroprotection. Here, we show that astrocyte mitochondrial uncoupling via viral overexpression of uncoupling protein 4 (UCP4) restores neuronal circuits and ion channel function in aged 3xTG AD mice with overt symptoms. Spontaneous local field potential recordings revealed a partial recovery of hippocampal and subicular sharp wave ripple oscillations, electrophysiological signatures of neuronal circuits known to be altered in AD. Combined whole-cell patch-clamp electrophysiology with two-photon Ca^2+^ imaging further demonstrated that UCP4 modulates activity-dependent Ca^2+^ influx, A-type potassium channel function, and enhances glial cell line-derived neurotrophic factor (GDNF) signaling. These findings identify astrocytic mitochondrial uncoupling as a potent mechanism enhancing neuronal resilience and restoring circuit function in symptomatic AD brains.

## 1. Introduction

Alzheimer’s disease (AD) is a progressive neurodegenerative disorder characterized by dysfunction of neurons across several brain regions, leading to cognitive decline and memory impairment. Astrocyte-to-neuron interactions have emerged as critical regulators of neuronal function and, when dysregulated, potential drivers of AD [1,2]. Upregulation of uncoupling protein 4 (UCP4) in mitochondria of astrocytes at pre-symptomatic stages of AD was found to be neuroprotective in a 3xTG AD mouse model [3]. UCP4 is a proton carrier located in the inner membrane of mitochondria, the activation of which uncouples oxidative phosphorylation from ATP synthesis [4,5]. The efficacy of mitochondrial uncoupling in older mice with overt AD phenotype as well as mechanisms behind observed neuroprotection remain to be evaluated.

Here, we investigated whether uncoupling of astrocyte mitochondria via overexpression of UCP4 protects subicular network function when initiated at symptomatic stages of AD and further explored the cellular and circuit-level mechanisms underlying this neuroprotection. Subiculum is the brain region anatomically located between the hippocampal CA1 and entorhinal cortex, involved in memory and spatial learning and shown to generate sharp wave ripple (SWR) oscillations [6,7]. SWRs are high-frequency brain oscillations critical for memory consolidation and spatial navigation [8,9,10]. Subiculum is the main output of the hippocampus critical for transfer of information from the hippocampal areas to cortex for memory consolidation [11]. Hence, proper function of subicular neurons is needed to ensure accurate perception and transmission of the SWR oscillations from the hippocampus to cortex to prevent learning and memory impairments [12]. Accordingly, compromised SWR dynamics were shown to disrupt memory consolidation, while their prolongation enhances it [13,14]. Hippocampal SWR oscillations are known to be impaired in AD, whereas subicular SWR events remain largely unstudied [15,16,17,18,19,20].

The generation of SWRs requires the coordinated activation of pyramidal cells and interneurons that display diverse firing behavior [21,22,23,24,25]. Among the ion channels tuning these neuronal firing patterns are voltage-gated Ca^2+^ (Cav) channels as well as Ca^2+^-sensitive, voltage-operated A-type K^+^ currents, which are of particular interest as they both have been implicated in the AD pathology [26,27].

Using ex vivo electrophysiological and imaging approaches, we found that upregulation of UCP4 corrects AD-related alterations in neuronal hippocampal–subicular microcircuits by modulating Ca^2+^ and K^+^ conductances and found evidence for the involvement of glial cell line-derived neurotrophic factor (GDNF) in these mechanisms. These findings establish uncoupling of astrocyte mitochondria as a potent mechanism increasing neuronal resilience and circuit protection in AD.

## 2. Materials and Methods

### 2.1. Animals

Male 3xTG AD mice (*B6;129-Tg(APPSwe,tauP301L)1Lfa Psen1tm1Mpm/Mmjax*) carrying three mutations associated with familial forms of Alzheimer’s disease and respective WT mice of *B6129SF2/J* background were used in the current study [28]. Mice were bred and housed in the animal facility of the University of Lausanne under a 12:12 light cycle (light on: 7:00–19:00) and fed with a regular maintenance diet (150 SP-25, Safe).

### 2.2. Viral Vector Construct and Delivery

We followed the same approach as previously [3] to achieve overexpression of UCP4 in mitochondria of astrocytes. Viral constructs, AAV9-GfaABC(1)D(B3)-mCherry:miR124T:WPRE (henceforth AAV-mCherry) and AAV9-GfaABC(1)D(B3)-mCherry:P2A:UCP4:3xHA-miR124T:WPRE (henceforth AAV-mCherry-UCP4) were custom produced by Vector Builder (Chicago, IL, USA). Astrocyte promoter GfaABC(1)D(B3) driven AAV viruses were used to achieve astrocyte specific expression of transgene. To suppress off-target transgene expression in neurons, a miR124T de-targeting sequence was incorporated [29]. p2A self-cleaving peptide was inserted to achieve co-expression of mCherry and UCP4 at similar levels.

6–7 months old 3xTG AD and WT mice were anesthetized with isoflurane (4% for induction, 12% for further maintenance) and then head-fixed on a stereotaxic frame. To reduce pain, the analgesic carprofen (5 mg/kg) was administered subcutaneously, while lidocaine (6 mg/kg) and bupivacaine (2.5 mg/kg) local anesthetics were injected under the scalp. A small cut was made to expose the skull, and two craniotomies per hemisphere were drilled above the viral injection sites. First injection coordinates were at anteroposterior (AP) −1 mm, mediolateral (ML) ±1.46 mm and ventral (V) −1.5 mm relative to bregma. Second injection coordinates were AP −1.56 mm, ML ±2.15 mm and V −1.5 mm relative to bregma. 500 nL of AAVmCherryUCP4 (titer of >2 × 10^12^ gc/mL) or AAV-mCherry (titer of >2 × 10^12^ gc/mL) viruses were infused at a rate of 100 nL/min using a Hamilton digital pump (KD Scientific, Harvard Bioscience, Inc., Holliston, MA, USA) and a thin glass pipette. To allow diffusion and prevent backflow of the virus, the glass pipette was left at the injection site for an extra 5 min. It was then slowly withdrawn over an additional 5 min period. After surgery completion, mice were placed in a heated chamber until full recovery of movement. The ophthalmic ointment was applied to prevent the eyes from drying during the surgery. To minimize pain sensitivity, paracetamol (2 mg/kg) was added to the drinking water for 3 days following the surgery.

### 2.3. Home-Cage Behavioral Assay

Digital ventilated cages (DVC, Tecniplast, Buguggiate, Italy) are automated home-cage monitoring units placed on a rack with electromagnetic sensor boards equipped with capacitance-sensing technology. 12 electromagnetic field-generating electrodes are evenly positioned in a 4 by 3 grid underneath each cage. Animal movement was recorded with a sampling rate of 4 Hz. Raw activity data are converted into multiple behavioral metrics, which are then extracted and analyzed using the DVC Analytics interface (Tecniplast).

To test whether 3xTG AD mice at age of 6–7 months exhibit disease-related deviations in their behavior, male 3xTG and WT mice were group housed (2–4 mice per cage) for 10 days prior to virus injection. We analyzed locomotor activity across the light and dark phases to probe for AD-associated disturbances in sleep–wake cycles. We also measured front/rear occupation metrics as a proxy for anxiety-like behavior.

### 2.4. Immunostaining and Confocal Microscopy

300 µm sagittal and 400 µm horizontal hippocampal slices were fixed with 4% PFA after electrophysiological measurements for further validation of virus expression. For immunohistochemistry, slices were permeabilized with 1% triton for 30 min and after-wards blocked in 2% normal horse serum for one hour. Brain slices were then preincubated in a blocking solution supplemented with primary antibodies for 2 days at 4 °C. After 2 days, samples were washed with PBS for 30 min and left overnight at 4 °C in the blocking solution containing secondary antibodies. Nuclei were labeled with Hoechst 33342 (1:1000; Invitrogen, Carlsbad, CA, USA). Fluorescent immunostaining was analyzed using a Leica Stellaris 8 confocal microscope (Leica, Wetzlar, Germany) equipped with 405 nm (UV), 488 nm (blue), 561 nm (green), and 638 nm (red) lasers and imaged using a 63×/1.4 PlanApo oil immersion objective. The following primary antibodies were used in the current study: mouse monoclonal anti-GFAP (1:300; G3893; Sigma-Aldrich, St. Louis, MO, USA), rabbit polyclonal anti-RFP (1:50; 600-401-379; Rockland, Limerick, PA, USA), mouse monoclonal anti-HSP70 (1:500; MA3-028; Invitrogen) and rabbit polyclonal anti HA (1:200; ab9110; Abcam, Cambridge, UK). The secondary antibodies used in this study were as follows: Alexa Fluor-488 goat anti-mouse (1:200; A11001; Invitrogen), Alexa Fluor-594 goat anti-rabbit (1:200; A32740; Invitrogen) and Alexa Fluor-647 goat anti-rabbit (1:200; A32733; Invitrogen).

### 2.5. Acute Brain Slice Preparation and Solutions

Mice were deeply anesthetized with isoflurane and then decapitated. The brain was isolated and placed in artificial cerebrospinal fluid (ACSF) containing (in mM): 124 NaCl, 26 NaHCO_3_, 3 KCl, 1.2 NaH_2_PO_4_, 2 MgSO_4_, 2 CaCl_2_ and 20 glucose, pH of 7.4 adjusted with 95% O_2_ and 5% CO_2_ carbogen and osmolarity of 298–300 mOsm/Kg. 300 µm sagittal or 400 µm horizontal hippocampal slices were prepared using a vibratome (VT1000S, Leica, Wetzlar, Germany). Brain slices were left to recover at 36.6 °C for 1 h and then kept at room temperature in the same ACSF before use for electrophysiology.

For further electrophysiological and imaging experiments, slices were placed in a heated perfusion chamber at 34 ± 1 °C and bathed in a recording ACSF solution containing (in mM): 119 NaCl, 26.2 NaHCO_3_, 2.5 KCl, 1 NaH_2_PO_4_, 1.3 MgSO_4_, 2.5 CaCl_2_ and 11 glucose, 0.01 DNQX, 0.01 picrotoxin, pH 7.4, bubbled with 95% O_2_/5% CO_2_ (osmolarity 300–310 mOsm/Kg). Chemicals used in the current study were obtained from Sigma-Aldrich and Tocris (Bio-Techne, Minneapolis, MN, USA) unless stated otherwise.

### 2.6. Extracellular Local Field Potential Recordings and Sharp Wave Ripple Analysis

Extracellular local field potentials (LFPs) were measured in recording ACSF solution without DNQX and picrotoxin, using glass electrodes with resistance of 0.2–0.5 MΩ and filled with the same ACSF. Electrodes were inserted in the pyramidal layer of hippocampal CA1 area and subiculum. LFPs were 200-fold amplified (Axon 700B; Molecular Devices LLC, San Jose, CA, USA) and digitized at 10 kHz (Axon Digidata 1550B; Molecular Devices LLC).

LFP recordings were bandpass filtered to 2.5–4000 Hz using Clampex 9.0 (Molecular Devices LLC) and further analyzed using modified code in MATLAB R2022b update 4 (9.13.0.2166757) (Natick, MA, USA) as described previously [13,30]. The MATLAB code is publicly available and can be found in https://github.com/GaifullinaAisylu/SWR-Average-Spectrograms/tree/main (accessed on 16 January 2026). For SWR detection, the signal was bandpass filtered in the ripple range (100–300 Hz; 4th-order Butterworth applied with zero-phase forward and filtfilt reverse filtering) and Hilbert transform was applied to identify the signal. The ripple envelope was obtained as the absolute value of the Hilbert signal. No smoothing was applied. Signals below the threshold of 3.5× the standard deviation of the envelope (computed over the entire analyzed segment), were excluded from the following analyses. SWRs with a duration of less than 10 ms and more than 200 ms were also excluded. Events with less than 15 ms interval between two of them were merged and analyzed as one event. To refine event boundaries in an SPW-like manner, 15 ms segments before and after the envelope were extracted and their median was computed. The onset and offset of the SWR/SPW complex were then refined as the first and last points at which the broadband LFP signal was below the local median within small search windows around the initial envelope defined boundaries. SWR/SPW duration was defined as the time between these selected boundaries, and SWR/SPW amplitude as the minimum LFP voltage within this window relative to the local median. The detected SWRs were later also confirmed by visual inspection. Time–frequency spectrograms were generated using a Fourier transform applied to 100 ms segments of ripple-filtered data centered on the SWR peak envelope, using a 15 ms sliding window with 90% overlap. Spectral power was estimated for frequencies between 100 and 300 Hz in 2 Hz steps. Recordings with no activity were discarded from the analysis.

### 2.7. Whole-Cell Patch-Clamp and Imaging

#### 2.7.1. Neuronal Spiking Activity

Neuronal spiking activity was measured in response to the injection of series of currents starting from −230 pA until 400 pA with a 30 pA increment for 320 ms. Whole-cell current-clamp measurements were recorded using an Axon 700B amplifier and ClampFit 9.0 software (Molecular Devices LLC). Data were digitalized with 2 kHz and filtered with Bessel Filter 10 kHz and analyzed using Clampex 9.0 software (Molecular Devices LLC).

Current-clamp recordings were executed in recording ACSF. Patch pipettes with a resistance of 3–5 MOhms were filled with an internal solution containing (in mM): 130 K-gluconate, 10 HEPES, 5 NaCl, 5 KCl, 1 MgCl_2_, 0.025 CaCl_2_, 0.1 EGTA, 4 glucose, 5 Na phosphocreatine, 4 Mg-ATP, 0.3 Na-GTP, pH of 7.3 adjusted with KOH and osmolarity of 290 mOsm/Kg.

To evaluate the effect of GDNF bath application on neuronal firing, neurons were clamped and recorded before and after 40 min bath application of 2 nM GDNF. To assess the effect of GDNF administration on the activity-related Ca^2+^ influx, brain slices were preincubated for at least 40 min in 2 nM GDNF before patching. Access resistance and resting membrane potential were monitored throughout recordings; recordings were excluded if variations of more than 25% or 5 mV, respectively, were detected.

#### 2.7.2. A-Type K^+^ Currents Recordings

Voltage-clamp recordings of A-type K^+^ currents were performed using the same pipette solution as above and slices were bathed in recording ACSF solution supplemented with 1 µM TTX. Two consecutive voltage-clamp protocols were used to isolate A-type K^+^ currents as described in [31]. Initially, neurons were held at −70 mV and pre-pulsed to −100 mV, and then 1000 ms depolarizing pulses from −70 mV to +20 mV with a 10 mV increment were applied with a 10 s inter-trace interval. Then, neurons were held at −70 mV, pre-pulsed to −100 mV for 1000 ms followed by stepping to −20 mV for a 200 ms after which 1000 ms depolarizing pulses from −70 mV to +20 mV with a 10 mV increment were applied with a 10 s inter-trace interval. To isolate A-type currents, neurons were pre-pulsed to −20 mV for 200 ms prior to application of depolarizing steps, which allowed elimination of fast-inactivating K^+^ currents (voltage-protocol described in Figure S3). A-type currents were obtained via digital current subtraction of second protocol from the first one. Currents were recorded using an Axon 700B amplifier and ClampFit 9.0 software (Molecular Devices LLC). Data were digitalized with 2 kHz and filtered with Bessel Filter 10 kHz and analyzed using Clampex 9.0 software (Molecular Devices LLC). Recordings were not corrected for liquid junction potential.

### 2.8. Two-Photon Ca^2+^ Imaging

Custom made 2-photon microscope was combined with whole-cell patch-clamp electrophysiology to study neuronal activity-related Ca^2+^ influx. Neurons were imaged using 20 × 1.0 NA long distance water-dipping objective (Olympus, Tokyo, Japan) and patched using a 3–4 MOhms glass pipette in recording ACSF solution. The same pipette solution as above was used with the addition of 20 µM Fluo2 K^+^ salt and 25 µM Alexa Fluor-594 Hydrazide to measure intracellular Ca^2+^ changes in somatic and dendritic compartments in response to 220 pA current injections. Fluorescence excitation (800 nm) was performed using a Chameleon Vision S femtosecond laser including group velocity dispersion compensation (Coherent, Glasgow, UK). Images were acquired at 2–3 frames per second over a field of view of 297 × 297 µm. Green and red signal outputs were detected at 525 ± 25 nm and 620 ± 30 nm, respectively. Image acquisition and analyses were performed using custom-written software in the LabVIEW 2013 (National Instruments, Austin, TX, USA) environment.

Somatic and dendritic Ca^2+^ signals were analyzed as previously described [32,33]. Briefly, regions of interest (ROIs) were selected to analyze somatic and proximal dendritic Ca^2+^ signals, respectively. Corresponding fluorescence values were then extracted separately from the green and red channels. The change in Ca^2+^ signal was calculated as

$$\frac{∆G}{R}=\frac{G-G0}{R}$$

where G is the fluorescence value from the green Ca^2+^-sensitive channel, while G0 is the baseline fluorescence value from the same channel before current pulse injection. R is the fluorescence value from the reference red channel.

### 2.9. GDNF Level Assessment

Astrocyte cultures were prepared from *B6129SF2/J* newborn pups (postnatal day PN 0–3) as described previously [34]. Astrocytes were isolated and cultured in Dulbecco’s Modified Eagle Medium (DMEM) supplemented with 10% heat-inactivated fetal bovine serum and 1% penicillin/streptomycin. After 14 days of culture, primary astrocytes were transduced with AAV-mCherry and AAV-mCherry-UCP4 viruses in 5:1000 dilution. Cells were used for GDNF-ELISA assay at 10 days post-infection.

Quantification of GDNF protein in astrocyte cell lysates was performed according to manufacturer’s guidelines (GDNF Mouse ELISA Kit, Abcam). In brief, cell lysates were prepared by homogenizing astrocytes in lysis buffer (137 mM NaCl, 20 mM TRIS pH 8, 1% NP40, 10% glycerol, 100 nM Na_3_VO_4_, Halt^TM^ Protease Inhibitor Single-Use Cocktail from Thermo Scientific (Waltham, MA, USA)). Cell lysates were then centrifuged for 10 min at 15,000× *g* at 4 °C and stored in ice-cooled-metal plate before use for ELISA. Cell lysate supernatants and respective standards were loaded in 96-well plate for further GDNF detection using Promega^TM^ GloMax Plate Reader (Promega, Madison, WI, USA). Biotinylated anti-mouse GDNF antibody and avidin-biotin-peroxidase complex were used for GDNF detection and signal amplification respectively. Samples were then treated with TMB color developing agent and the assay was terminated by addition of the TMB stop solution. The O.D. absorbance was read at 450 nm. GDNF levels were normalized to total protein and expressed as picograms per mg. The assay sensitivity ranges between 10 and 2000 pg/mL.

### 2.10. Statistics and Data Analysis

Statistical analyses were performed in GraphPad Prism 9 software. Outliers were identified and removed using a ROUT method. Statistical difference was tested using nonparametric Mann–Whitney and Wilcoxon tests and 2-way ANOVA followed by Fisher’s LSD test. Note that this post hoc test commonly used in this type of studies with small factorial designs does not formally control family-wise Type I error when multiple simple effects are tested. Statistical significances are according to two-tailed * *p* < 0.05, ** *p* < 0.01, *** *p* < 0.001, and **** *p* < 0.0001. Details on statistical comparisons are provided in Tables S1–S13.

## 3. Results

### 3.1. Selective Expression of UCP4 in Astrocyte Mitochondria

**Figure 1:**
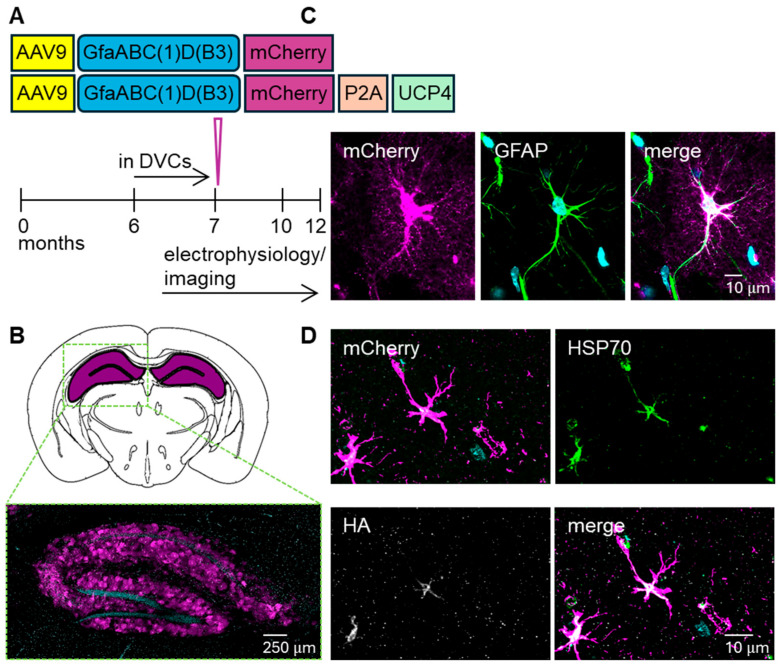
Characterization of viral vectors used to drive UCP4 expression in astrocytic mitochondria. (**A**) Design of AAV vectors used to infect astrocytes and experimental timeline. Prior to viral vector injection at 6–7-month of age, mouse behavior in home cages (DVCs) was performed. Functional assessments by electrophysiology and Ca^2+^ imaging were performed at 10–12 months of age. (**B**) Transgene expression was detected in astrocytes across the entire hippocampal formation. (**C**) Confocal images showing overlap of mCherry signal and GFAP immunostaining. (**D**) Confocal images showing overlap of astrocytic mCherry signal (**top left**) with mitochondrial marker HSP70 (**top right**), and the UCP4 HA-tag (**bottom left**), together with the merged image (**bottom right**).

To elevate the levels of UCP4 in astrocytes, we injected adeno-associated viruses that specifically target mitochondria of astrocytes, namely a vector expressing mCherry only under the GfaABC(1)D(B3) promoter, which was used as a control, and a vector expressing mCherry along with UCP4 (Figure 1A), as described previously [3]. Injection of AAV-mCherry-UCP4 and respective AAV-mCherry control viral vector (Figure 1A) resulted in a transgene expression spreading across the dentate gyrus, hippocampus, and subiculum (Figure 1B).

Furthermore, transgene expression was found in astrocytes, as demonstrated by the overlap of mCherry fluorescence signal with glial fibrillary acidic protein (GFAP) immunostaining (Figure 1C). Colocalization studies of mitochondrial heat shock protein 70 (HSP70) immunostaining with UCP4-HA tag immunostaining demonstrated successful expression of UCP4 in mitochondria (Figure 1D). This was consistent with our previous study, where we showed, using qRT-PCR, a 1.25-fold increase in UCP4 mRNA transcript levels upon overexpression [3].

### 3.2. AD-Related Phenotype Characterization Using Home-Cage Behavioral Analyses

Viral vector-mediated overexpression of mitochondrial UCP4 in astrocytes of AD mice administered at 2 months (pre-symptomatic stage of disease) and recorded at 7–10 months prevented neuronal degeneration and preserved spatial memory [3]. As clinical diagnosis of AD typically occurs when its symptoms become apparent, we next aimed to test whether UCP4 treatment applied in older mice with overt AD symptoms was also effective.

**Figure 2:**
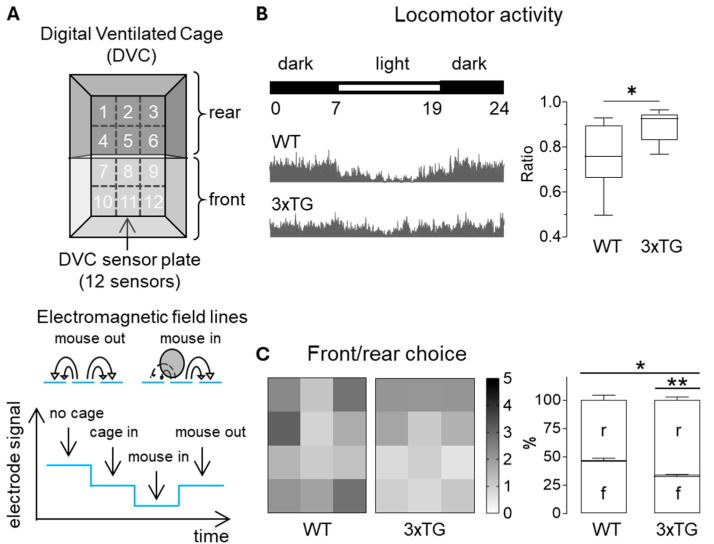
Home-cage detection of AD-related alterations in mouse behavior. (**A**) DVCs are positioned on racks containing electromagnetic sensor boards that use capacitance-based detection. The sensor board contains 12 electrodes detecting electromagnetic field variations upon animal movement, which are used as read-outs of animal activity. The circle represents a mouse and the lines are sued to represent the change in conductance as the mouse passes over the electrodes. Schemes were modified from Klein et al. [37]. (**B**) Representative actograms of dark/light phase activity of WT and 3xTG AD mice across 24 h cycles within a single day (**left**). WT mice were more active during the dark phase, whereas 3xTG AD mice showed similar activity levels across light and dark phases. Data are presented as ratio of light/dark phase activity of WT (*n* = 21 mice, *N* = 9 cages, 10 days of continuous measurement) and 3xTG AD mice (*n* = 14 mice, *N* = 6 cages) (**right**). * *p* < 0.05 (Mann–Whitney). Data are presented as box and whiskers (min to max) plots. (**C**) Heat maps of front/rear choice of WT and 3xTG AD mice (**left**) over the analyzed 10 days and front (f) vs. rear (r) occupation metrics (**right**), indicating that 3xTG AD mice spent more time in the rear zone of the cage. Additional details of the statistical comparisons are provided in Table S1. Data are percentage of front/rear cage zones occupation for WT (*n* = 21 mice, *N* = 9 cages, 10 days of continuous measurement) and 3xTG AD mice (*n* = 14 mice, *N* = 6 cages). * *p* < 0.05, ** *p* < 0.01 (Chi^2^ and Mann–Whitney test respectively). Data are presented as mean ± SEM.

Prior stereotaxic viral injection, 6–7 months of age male WT and 3xTG mice were group housed in DVCs, a platform that allows continuous and noninvasive monitoring of animal behavior in a home-cage environment (Figure 2A). Using the DVC system, we quantified animal locomotor activity across light and dark phases as well as front/rear occupation, metrics commonly used to characterize disturbances in sleep and anxiety-like behavior in AD [35,36]. As anticipated, home-cage behavior analyses revealed that 3xTG AD mice did not show preferred phase activity, while WT mice were predominantly active during the dark phase (Figure 2B). The latter suggests a disruption of both circadian rhythms and sleep in AD mice. To further assess anxiety-like behavior, heat maps of front and rear cage zone occupancy were analyzed. As shown in Figure 2C, 3xTG AD mice spent more time in the rear zone of the cage, exhibiting an avoidance pattern, characteristic of increased anxiety-like behavior. Overall, these metrics indicated that AD-associated alterations are behaviorally detectable in 6–7-months-old 3xTG AD mice.

### 3.3. Overexpression of UCP4 in Astrocyte Mitochondria Partially Restored Hippocampal-Subicular Oscillations in AD Mice

**Figure 3:**
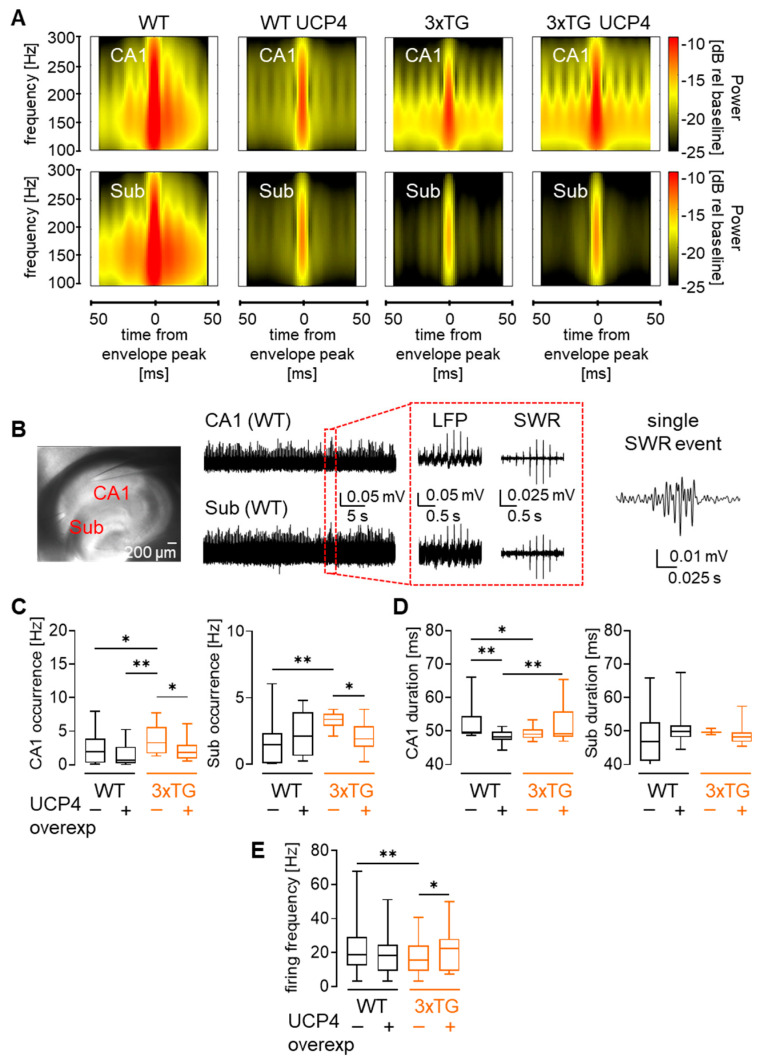
Neural network activity in AD mice without and with UCP4 treatment. (**A**) Average time–frequency power spectrum of LFPs recorded from hippocampus and subiculum (0–50 ms bins). Events were aligned at their peak amplitude and displayed in the ±50 ms range. The color-coded power amplitude scale (dB) is shown. (**B**) Schematic representation of extracellular LFP recordings. Representative raw and filtered LFP traces for SWR extraction and a single SWR event. (**C**) Frequency of SWR events (occurrence, Hz) recorded from the CA1 and subiculum. 3xTG AD mice exhibited a higher event frequency compared to WT mice, which was corrected by UCP4 overexpression. 2-way ANOVA analyses of occurrence of hippocampal CA1 SWRs showed significant effects of genotype (F_(1, 61)_ = 4.723, *p* = 0.0336) and UCP4 treatment (F_(1, 61)_ = 4.808, *p* = 0.0321) with no interaction between these factors (F_(1, 61)_ = 0.7295, *p* = 0.3964). On the contrary, 2-way ANOVA analyses of occurrence of subicular SWR events did not reveal significant effects of genotype (F_(1, 57)_ = 2.988, *p* = 0.0893) and UCP4 treatment (F_(1, 57)_ = 0.4930, *p* = 0.4854) but a significant interaction of these factors (F_(1, 57)_ = 5.888, *p* = 0.0184). * *p* < 0.05, ** *p* < 0.01 (by Fisher’s LSD post hoc test). Details of the statistical comparisons are provided in Table S2. Data are occurrence (Hz) for CA1 WT *n* slices = 14, *N* mice = 5; WT UCP4 *n* slices = 24, *N* mice = 5; 3xTG AD *n* slices = 15, *N* mice = 6; 3xTG UCP4 *n* slices = 12, *N* mice = 3; subiculum WT *n* slices = 14, *N* mice = 5; WT UCP4 *n* slices = 23, *N* mice = 5; 3xTG AD *n* slices = 12, *N* mice = 5; 3xTG UCP4 *n* slices = 12, *N* mice = 3. (**D**) Duration of SWR events recorded from CA1 and subiculum in the four experimental groups. SWR dynamics revealed a shorter SWR event duration in 3xTG AD compared to WT that was rescued in 3xTG AD mice overexpressing UCP4 in CA1 hippocampal region. 2-way ANOVA analyses of duration of CA1 SWRs did not reveal significant effects of genotype (F_(1, 58)_ = 0.1025, *p* = 0.75) and UCP4 treatment (F_(1, 58)_ = 0.3543, *p* = 0.554) but a significant interaction of these factors (F_(1, 58)_ = 11.03, *p* = 0.0016). 2-way ANOVA analyses of duration of subicular SWRs did not show significant effects of genotype (F_(1, 57)_ = 0.005113, *p* = 0.9432), UCP4 treatment (F_(1, 57)_ = 0.6606, *p* = 0.4197) and no significant genotype- interaction between these factors (F_(1, 57)_ = 2.377, *p* = 0.1287). * *p* < 0.05, ** *p* < 0.01 (by Fisher’s LSD post hoc test). Additional details of the statistical comparisons are provided in Table S2. Data are durations (ms) for CA1 WT *n* slices = 11, *N* mice = 5; WT UCP4 *n* slices = 24, *N* mice = 5; 3xTG AD *n* slices = 15, *N* mice = 6; 3xTG UCP4 *n* slices = 12, *N* mice = 3; subiculum WT *n* slices = 14, *N* mice = 5; WT UCP4 *n* slices = 23, *N* mice = 5; 3xTG AD *n* slices = 12, *N* mice = 5; 3xTG UCP4 *n* slices = 12, *N* mice = 3. (**E**) Neuronal action potential firing frequency at 220 pA current injection, measured by whole-cell patch-clamp, showing a reduction in firing frequency of 3xTG AD neurons compared to WT, and recovery of frequency following UCP4 overexpression. 2-way ANOVA test revealed significant genotype-treatment interaction (F_(1, 287)_ = 4.589, *p* = 0.0330), whereas neither genotype (F_(1, 287)_ = 0.09115, *p* = 0.7629) nor treatment (F_(1, 287)_ = 0.6279, *p* = 0.4288) alone showed significant effects. * *p* < 0.05, ** *p* < 0.01 (by Fisher’s LSD post hoc test). Additional details of the statistical comparisons are provided in Table S2. Data are firing frequency (Hz) for WT *n* neurons = 135, *N* mice = 29; WT UCP4 *n* neurons = 28, *N* mice = 7; 3xTG AD *n* neurons = 102, *N* mice = 38; 3xTG UCP4 *n* neurons = 26, *N* mice = 6. Data are presented as box and whiskers (min to max) plots.

After confirming the presence of AD symptoms in 3xTG AD mice at 6–7 months of age, we proceeded with stereotaxic viral injections resulting in four groups of animals: WT AAV-mCherry (WT), WT AAV-mCherry-UCP4 (WT UCP4), 3xTG AAV-mCherry (3xTG), 3xTG AAV-mCherry-UCP4 (3xTG UCP4). After 3 months of post-injection, we assessed high-frequency brain network oscillations crucial for memory function by performing extracellular LFP recordings on horizontal hippocampal slices. Time–frequency spectrogram plots (Figure 3A) revealed oscillatory activity in the 100–300 Hz range within the hippocampus and subiculum. We then quantified the frequency of occurrence and duration of high-frequency SWR events, as shifts in these parameters correlate with changes in memory performance (Figure 3B). In line with previous reports [38], frequency of occurrence of SWR events (Figure 3C) was increased by ~2-fold in 3xTG AD mice compared to WT. This increase suggests altered network dynamics due to a shift in synaptic excitation–inhibition balance toward increased excitability, thereby resulting in more frequent SWR events, as reported in a previous study [38]. Disease-associated increase in the occurrence of SWR events was reduced by UCP4 overexpression in CA1 regardless of genotype, whereas in subiculum, the effect of UCP4 was selective (Figure 3C).

We next investigated the duration of SWR events which were shown to be critical for memory facilitation, with long-duration ripples supporting more extensive network replay and stronger memory formation [13]. We observed that hippocampal but not subicular SWR event durations were shorter in 3xTG AD slices compared to WT (Figure 3D). Slices from 3xTG AD mice overexpressing UCP4 showed a modest but significant 1.1-fold increase in the duration of hippocampal SWR events, suggesting an improved network coordination in mice with established AD symptoms. On the other hand, UCP4 overexpression in WT mice did not significantly affect occurrence, but it decreased the duration of CA1 SWR oscillations.

Change in the network coordination often suggests shifts in neuronal excitability. Subiculum is the brain region affected by AD among the first, yet remains relatively understudied compared to the CA1 hippocampal region. We therefore focused on subicular neurons to further address AD- and UCP4-mediated changes in neuronal function. Subicular neurons are known to display burst, doublet, and regular firing patterns [39]. Accordingly, we found the three types of firing profiles in whole-cell current-clamped subicular neurons (Figure S1A,B). We next assessed firing frequency evoked by 220 pA current step injection in the pooled population of neurons from WT and 3xTG AD, which revealed an overall lower neuronal firing frequency in 3xTG AD mice compared to WT. This observed difference, however, was corrected by UCP4 overexpression (Figure 3E). In contrast, UCP4 overexpression had no significant effect on the firing frequency in WT mice. For analysis of firing frequency, neurons with different firing profiles were pooled to assess the overall effect at the population level; no further subgroup-region specific comparisons were performed. To increase statistical power, data recorded from WT and 3xTG AD mice injected with AAV-mCherry control were merged with the data recorded from naïve WT and 3xTG AD mice as no significant differences were found between these two conditions in the proportion of burst vs. regular spiking neurons (Figure S1B), in both passive and active electrophysiological properties (Figure S2). The latest data also indicates that viral transduction itself does not alter neuronal intrinsic properties. In conclusion, overexpression of UCP4 partially restored AD-related alterations in hippocampal–subicular microcircuits and in the firing frequency of individual neurons of 3xTG AD mice. These results suggest that the astrocytic mitochondrial uncoupling treatment remains beneficial for brain function when started after the onset of detectable AD-associated symptoms.

### 3.4. Overexpression of UCP4 Enhances Neuronal Activity-Related Ca^2+^ Influx

To address cellular mechanisms underlying UCP4 mediated modulation of firing frequency, we analyzed ion channels shaping this activity, among which are A-type potassium and Cav channels.

**Figure 4:**
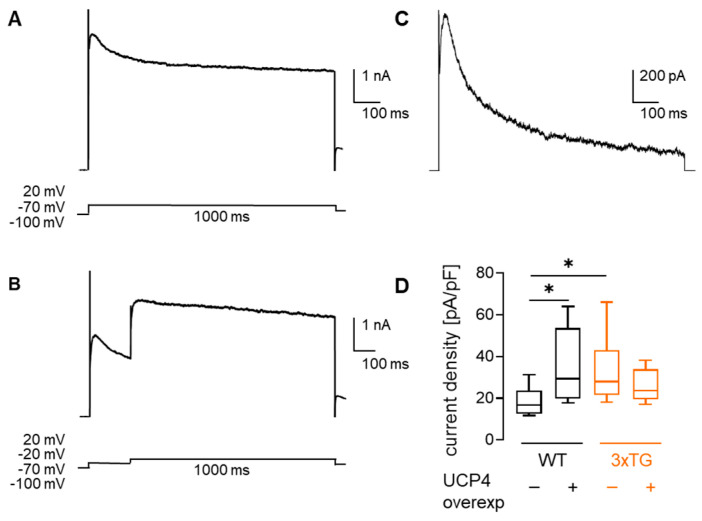
Mild mitochondrial uncoupling affects A-type K^+^ currents in subicular neurons of 3xTG AD mice. (**A**) Outward potassium currents elicited by stepping voltage-clamped neurons from −100 mV to +20 mV for 1000 ms. (**B**) To reveal A-type currents, fast-inactivating K^+^ current components were diminished by pre-pulsing neurons to −20 mV for 200 ms before stepping to +20 mV. (**C**) A-type currents were obtained via subtraction of sustained current (**B**) from the total current (**A**). (**D**) Quantification of A-type K^+^ current density (pA/pF) at +20 mV in the four experimental groups. 2-way ANOVA test revealed significant genotype-treatment interaction (F_(1, 27)_ = 6.016, *p* = 0.0209), although neither genotype (F_(1, 27)_ = 0.2456, *p* = 0.6242) nor treatment (F_(1, 27)_ = 0.9286, *p* = 0.3438) showed significant main effects. * *p* < 0.05 (by Fisher’s LSD post hoc test). Additional details of the statistical comparisons are provided in Table S3. WT *n* neurons = 8, *N* mice = 3; WT UCP4 *n* neurons = 8, *N* mice = 2; 3xTG AD *n* neurons = 9, *N* = 4; 3xTG AD UCP4 *n* neurons = 6, *N* mice = 2. Data are presented as box and whiskers plots.

Whole-cell voltage-clamp protocols were used to extract A-type K^+^ currents (Figure 4A–C) as described previously [31]. Assessment of K^+^ current density recorded from subicular neurons revealed a 1.7-fold larger A-type currents in 3xTG AD mice compared to WT controls (Figure 4D), while UCP4 overexpression prevented this increase. On the other hand, larger A-type K^+^ currents were observed in WT mice overexpressing UCP4. Thus, UCP4 exerts a genotype-dependent effect on A-type K^+^ currents, with current density in 3xTG AD mice overexpressing UCP4 no longer significantly differing from WT. However, the direct comparison of current densities between 3xTG and 3xTG UCP4 did not reach statistical significance, suggesting a partial rather than a statistically confirmed rescue.

A-type K^+^ currents are known to be modulated by cellular redox state [40,41]. As uncoupling proteins are key regulators of mitochondrial reactive oxygen species production and cellular redox balance, we aimed to explore whether UCP4 overexpression could normalize A-type K^+^ currents function via modulation of oxidative stress. Cellular dialysis of the antioxidant agent glutathione, however, failed to reveal signs of oxidative dysfunction of A-type K^+^ currents in subicular neurons in 3xTG AD compared to WT mice (Figure S3).

**Figure 5:**
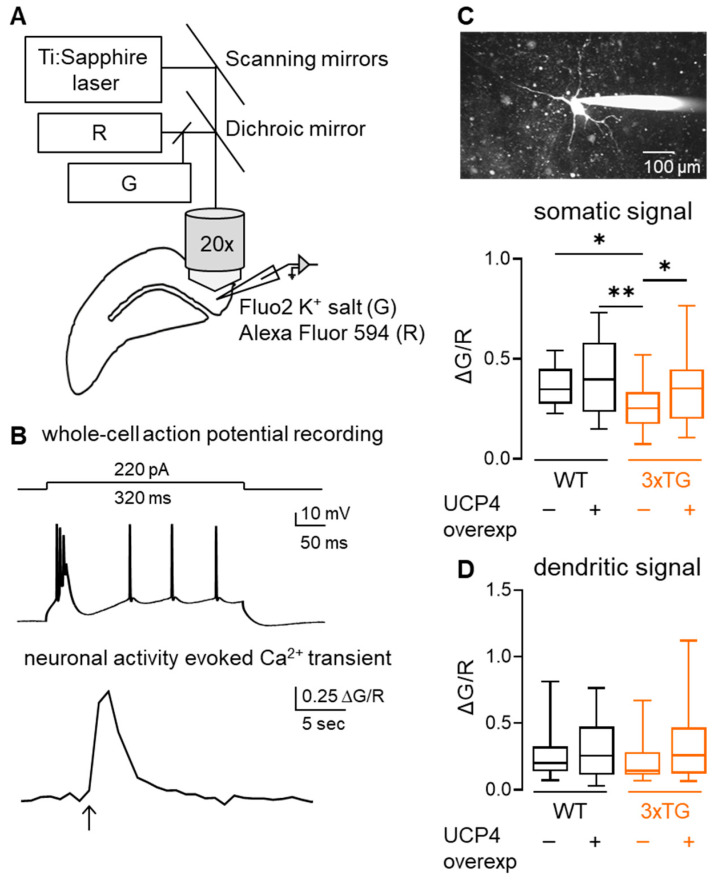
Overexpression of UCP4 enhanced activity-dependent Ca^2+^ influx in subicular neurons of 3xTG AD mice. (**A**) Schematic representation of 2photon Ca^2+^ imaging combined with whole-cell patch-clamp electrophysiology. (**B**) Current-clamp action potentials (mV) evoked by 220 pA current injection and respective Ca^2+^ transient (ΔG/R). The arrow indicates the starting point of the Ca^2+^ signal (**C**) An image of whole-cell neuron filled with Fluo2 K^+^ salt and Alexa Fluor 594 (**top**). Quantification of somatic Ca^2+^ responses to neuronal firing (**below**). Ca^2+^ transients from 3xTG AD neurons were of lower amplitude than those of WT. UCP4 overexpression rescued somatic Ca^2+^ transients in 3xTG AD mice. 2-way ANOVA analyses of activity-dependent Ca^2+^ transients showed significant effects of genotype (F_(1, 76)_ = 4.716, *p* = 0.033) and UCP4 treatment (F_(1, 76)_ = 5.100, *p* = 0.0268) with no interaction between these factors (F_(1, 76)_ = 0.6349, *p* = 0.428). * *p* < 0.05, ** *p* < 0.01 (by Fisher’s LSD post hoc test). Additional details of the statistical comparisons are provided in Table S4. WT *n* neurons = 20, *N* mice = 12; WT UCP4 *n* neurons = 17, *N* mice = 5; 3xTG AD *n* neurons = 29, *N* mice = 14; 3xTG UCP4 *n* neurons = 14, *N* mice = 4. (**D**) Quantification of proximal dendritic Ca^2+^ responses to neuronal firing. Dendritic Ca^2+^ transients displayed the same trend as the somatic counterparts, without reaching significantly different values between groups. 2-way ANOVA analyses of activity-dependent dendritic Ca^2+^ transients did find significant effects of genotype (F_(1, 70)_ = 0.03710, *p* = 0.8478) and UCP4 treatment (F_(1, 70)_ = 3.090, *p* = 0.0831) with no interaction between these factors (F_(1, 70)_ = 0.7452, *p* = 0.3909). Additional details of the statistical comparisons are provided in Table S4. Data for WT *n* neurons = 18, *N* mice = 12; WT UCP4 *n* neurons = 17, *N* mice = 5; 3xTG *n* neurons = 27, *N* mice = 15; 3xTG UCP4 *n* neurons = 12, *N* mice = 4. Data are presented as box and whiskers (min to max) plots.

Voltage-gated Ca^2+^ channels open upon membrane depolarization and cause Ca^2+^ influx from the extracellular compartment leading to a change in cytosolic Ca^2+^ levels. To test whether the observed increase in neuronal firing frequency correlates with larger Ca^2+^ influx, 2-photon Ca^2+^ imaging combined with whole-cell patch-clamp recordings of neuronal activity were performed (Figure 5A,B). Neurons from 3xTG AD mice displayed lower somatic Ca^2+^ transients than neurons of WT mice (Figure 5C). UCP4-mediated mild uncoupling of astrocyte mitochondria led to a 1.4-fold increase in somatic Ca^2+^ influx in subicular neurons of 3xTG AD mice, comparable to values observed in WT neurons (Figure 5C). On the contrary, upregulation of UCP4 in WT mice did not yield significant changes in somatic Ca^2+^ transients. Neither genotype or treatment significantly affected dendritic Ca^2+^ transients (Figure 5D). The neuronal firing pattern (bursting vs. regular spiking) did not significantly affect the recorded Ca^2+^ transients (Figure S4).

The reduction in the activity-related Ca^2+^ influx observed in 3xTG AD mice suggests disease-associated dysfunction of Cav channels. In subicular neurons, low-voltage activated (LVA) Ca^2+^ currents are generated by T-type Ca^2+^ channels that support subicular burst firing crucial for learning and memory [42,43]. Accordingly, whole-cell voltage-clamp recordings revealed substantial LVA Ca^2+^ currents in subicular neurons of AD mice that were suppressed by the T-type channel blocker Z944 (Figure S5A). Blocking LVA Ca^2+^ conductances by Z944 prevented the generation of action potential bursts resulting in a conversion to a regular firing pattern (Figure S5B). We found that LVA currents differed in 3xTG AD neurons compared to WT, showing a reduced amplitude. However, this difference did not reach significance when normalized to membrane capacitance (i.e., current density). LVA currents also display faster inactivation time constant kinetics in 3xTG AD mice (Figure S5C). Interestingly, a shift towards more positive potentials was detected in the steady-state activation of LVA Ca^2+^ currents, leading to reduced window currents in 3xTG AD mice (Figure S5D). Window currents represent a steady-state Ca^2+^ influx occurring in a range of voltages where Cav channels’ inactivation and activation curves overlap and are critical for maintaining intracellular Ca^2+^ homeostasis [44]. We further addressed the effect of UCP4 on neuronal T-type Ca^2+^ channel-mediated Ca^2+^ transients in subicular neurons of the four experimental groups before and after application of Z944. These experiments did not reveal an increase in either somatic or dendritic T-type Ca^2+^ channel-mediated Ca^2+^ transients between 3xTG and 3xTG-UCP4 mice (Figure S6A), which suggests that UCP4 mediated increase in the activity-related Ca^2+^ influx in 3xTG AD mice was not driven by T-type Ca^2+^ channels. Finally, we tested the potential involvement of ryanodine receptors (RyR), since Ca^2+^ release from internal stores via RyR are often described to be altered in AD mouse models [45]. However, the Ca^2+^ signal caused by application of caffeine, a direct RyR agonist, was not different between subicular neurons of 3xTG AD and WT mice (Figure S6B).

This set of experiments indicated that 3xTG AD mice display larger A-type potassium currents, which may contribute to the reduced somatic firing frequency observed in this model. UCP4 upregulation exerts a genotype-dependent effect on A-type current density, shifting values in 3xTG AD mice toward those observed in WT mice. The measurements also showed that neurons from the 3xTG AD mice displayed decreased activity-related Ca^2+^ influx, which was reversed by UCP4 overexpression.

### 3.5. Overexpression of UCP4 Upregulates GDNF Levels

**Figure 6:**
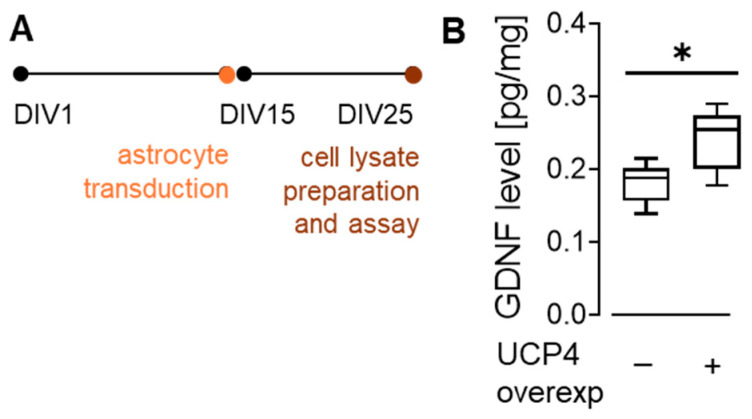
UCP4 overexpression increased GDNF. (**A**) Timeline of experiments. (**B**) Quantification of GDNF by ELISA was performed on cell lysates obtained from primary astrocyte cultures transduced with either AAV-mCherry (control, *n* samples = 6, *N* mice = 6) or AAV-mCherry-UCP4 (*n* samples = 7, *N* mice = 7) for 10 days before GDNF level assessment. GDNF levels (pg/mg) for control and UCP4. * *p* < 0.05 (Mann–Whitney). Details of the statistical comparisons are provided in Table S5. Data are presented as box and whiskers (min to max) plots.

Bath application of GDNF was shown to both increase Cav channel activity and reduce A-type K^+^ currents in dopaminergic neurons [46]. Thus, those described changes driven by GDNF application closely resemble the effects observed following UCP4 overexpression shown above. We therefore hypothesized that UCP4 overexpression in astrocytes may enhance GDNF production. To test this hypothesis, we quantified GDNF levels in primary astrocytes transduced with either AAV9-mCherry-UCP4 or control AAV9-mCherry viral vectors (Figure 6A). We found that UCP4 overexpression in astrocytes enhanced their production of GDNF by 1.3-fold compared to controls (Figure 6B). This result supports the notion that GDNF mediates the link between astrocytic UCP4 overexpression and the modulation of neuronal function.

### 3.6. GDNF Increases Neuronal Firing Frequency and Activity-Related Ca^2+^ Influx in 3xTG AD Mice

**Figure 7:**
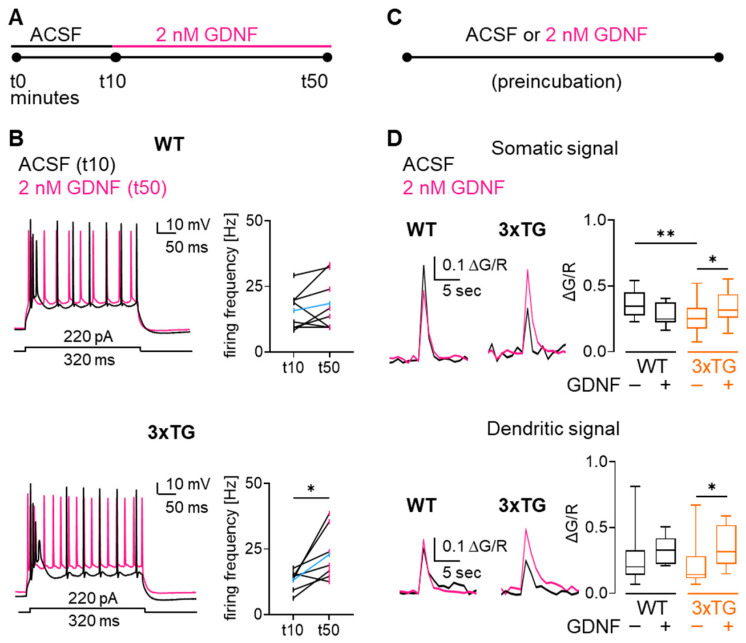
GDNF accelerated neuronal firing frequency and increased activity-related Ca^2+^ transients in 3xTG AD mice. (**A**) Experimental design of acute GDNF bath application. (**B**) WT and 3xTG AD mice current-clamp traces elicited by 220 pA current injection in subicular neurons before (black) and after (pink) 40 min bath application of 2 nM GDNF (**top left**). Quantification of action potential firing frequency of WT and 3xTG AD mice subicular neurons before (t10, black) and after (t50, pink) GDNF (**top right**). Acute GDNF did not affect neuronal firing frequency in WT mice (n neurons = 8, N mice = 5; Wilcoxon test). On the contrary, GDNF significantly enhanced neuronal firing frequency in 3xTG AD mice (**right**) (n neurons = 7, N mice = 4; * *p* < 0.05, Wilcoxon test). Mean values are indicated in blue. (**C**) Experimental design depicting that brain slices were preincubated in GDNF for at least 40 min before current-clamp measurements. (**D**) Original traces and quantification of transient amplitude (ΔG/R) of WT and 3xTG AD somatic (**top**) and dendritic (**bottom**) Ca^2+^ transients in the absence (black) and in the presence (pink) of 2 nM GDNF. Somatic, but not dendritic, Ca^2+^ transients were reduced in 3xTG AD compared to WT mice. In both regions, GDNF increased Ca^2+^ transients from 3xTG AD. 2-way ANOVA analyses of activity-related somatic Ca^2+^ transients revealed a significant genotype-treatment interaction (F_(1, 68)_ = 7.660, *p* = 0.0073), while the main effects of genotype (F_(1, 68)_ = 0.4486, *p* = 0.5053) and treatment (F_(1, 68)_ = 0.002787, *p* = 0.9581) were not significant. 2-way ANOVA analyses of activity-related dendritic Ca^2+^ transients revealed significant effect of treatment (F_(1, 59)_ = 4.022, *p* = 0.0495), while no differences were observed in genotype (F_(1, 59)_ = 0.02269, *p* = 0.8808) and interaction effects (F_(1, 59)_ = 0.8265, *p* = 0.367). * *p* < 0.05, ** *p* < 0.01 (by Fisher’s LSD post hoc test). Additional details of the statistical comparisons are provided in Table S6. WT somatic/dendritic ΔG/R *n* neurons = 20/18, *N* mice = 12/12; WT GDNF somatic/dendritic ΔG/R *n* neurons = 8/5, *N* mice = 3/3; 3xTG somatic/dendritic ΔG/R *n* neurons = 29/27, *N* mice = 14/15 mice; 3xTG GDNF somatic/dendritic ΔG/R *n* = 15/13 neurons, *N* mice = 6/6. Data are presented as box and whiskers plots.

We next investigated the functional effect of GDNF on neuronal firing frequency and activity-related Ca^2+^ influx in WT and 3xTG AD mice. To assess the impact of acute GDNF bath application on neuronal firing, subicular neurons were recorded in whole-cell configuration before and after 40 min of bath application of 2 nM GDNF (Figure 7A). Acute bath application of GDNF enhanced neuronal firing frequency evoked by 220 pA current injection in 3xTG by 1.7-fold, while it had no significant effect on the subicular neurons of WT mice (Figure 7B). To verify that such long recordings would not cause unwanted drifts in neuronal firing due to compromised cellular integrity or dilution of cytosolic components, we carried out long-term whole-cell recordings in ACSF without GDNF (Figure S7). These experiments did not reveal significant changes in the firing frequency of subicular neurons from either 3xTG or WT mice, confirming that the observed increase in firing frequency induced by GDNF was not attributable to technical limitations.

To evaluate the effect of GDNF exposure on activity-dependent Ca^2+^ influx in subicular neurons, brain slices were preincubated for 40 min in 2 nM GDNF prior to electrophysiological recordings and compared with cells incubated in control ACSF (Figure 7C).

Whole-cell recordings combined with 2-photon Ca^2+^ imaging yielded 1.2-fold and 2.1-fold increase in somatic and dendritic Ca^2+^ transients, respectively, in 3xTG AD mice. GDNF administration did not have a significant effect on somatic and dendritic Ca^2+^ transients in WT mice (Figure 7D). Collectively, these findings position GDNF as a plausible intermediate linking astrocytic mitochondrial UCP4 and the neuronal function.

## 4. Discussion

As neuron-based therapies for AD repeatedly face setbacks, attention is increasingly shifting toward non-neuronal cells such as astrocytes, microglia, and oligodendrocytes. We recently demonstrated that overexpression of mitochondrial UCP4 in astrocytes of pre-symptomatic AD mice prevented multilevel dysfunctions, including spatial memory impairments [3]. However, most AD patients start receiving treatments at advanced stages of the disease. The present study aimed to establish whether UCP4 treatment remains effective when initiated in older mice with overt AD symptoms and to probe the cellular and circuit-level mechanisms underlying this neuroprotection.

Mitochondrial uncoupling proteins function as proton carriers allowing proton flow from the intermembrane space into the matrix resulting in mild depolarization of the mitochondrial membrane potential [5,47]. The UCP4 isoform is predominantly expressed in the brain and, importantly, is known to decline in AD patients [4,48,49]. It was shown that acting on mitochondrial UCP4 in astrocytes as well as in neurons leads to complex changes in cellular metabolism [5,47]. For example, overexpression of UCP4 in astrocytes shifted energy production from oxidative phosphorylation towards glycolysis resulting in improved neuronal function in vitro potentially through enhanced lactate production and decreased mitochondrial reactive oxygen species production [5]. Upregulation of UCP4 in vivo in astrocytes of 3xTG AD mice prevented disease-related changes in brain metabolites as well as in memory performance [3]. Overall, astrocyte mitochondria emerge as a critical regulator of astrocyte-to-neuron metabolic interactions and potent modulator of neuronal behavior in health and disease [3,5,50].

In a first phase, we aimed to test whether overexpression of mitochondrial UCP4 in astrocytes improves network brain oscillations involved in memory consolidation and spatial navigation in 3xTG AD mice [28] with established AD symptoms. We utilized a home-cage monitoring system to establish the presence of disease-related deviations in 3xTG AD mice behavior at the age of 6 to 7 months. Assessment of animal activity during the light–dark phases of the 24 h cycle showed that 3xTG AD mice exhibited comparable level of activity during the light–dark phases suggesting disruptions in circadian rhythms and sleep–wake cycles. In addition, 3xTG AD mice had a preference for the dark rear area of the cage exhibiting avoidance patterns. These behavioral features mimic AD-related changes in sleep and anxiety-related behavior [35,36,51].

We then recorded and analyzed high-frequency SWR brain activity in acute hippocampal slices from mice 3 months after viral vector injection. SWR oscillations are well-established electrophysiological signatures of memory consolidation and retrieval. Disruptions in SWR dynamics serve as an early indicator of network dysfunction and cognitive impairment in AD, while their prolongation was shown to improve memory [13,52]. Analysis of SWR dynamics revealed pathology-related differences between 3xTG AD and WT mice at the level of SWR event frequency and duration. We found that UCP4 overexpression led to the prolongation of SWR events and decreased their occurrence in CA1 hippocampal region of 3xTG AD mice, while significantly shortening SWR duration in WT mice, bringing it closer to the levels observed in 3xTG AD mice. UCP4 overexpression reduced the occurrence of SWRs in the subiculum of 3xTG AD mice without altering their duration. Taken together, these findings link UCP4 upregulation in astrocytes to enhanced local neural network activity in AD. This circuit-level effect of UCP4, not described before, is of importance as emerging research suggests a direct link between improved local circuit activity and higher memory performance [53,54,55].

The improvement in local network activity caused by UCP4 treatment pointed towards changes in neuronal function in AD. To test this hypothesis, we performed whole-cell current-clamp action potential measurements on subicular neurons. We found that UCP4 overexpression in 3xTG AD mice corrected disease-related reduction in neuronal firing. As neuronal firing is modulated by a variety of ion channels, we investigated candidate channels underlying this activity. A-type Kv4 currents shape neuronal firing via their effects on the time interval between spikes [56,57,58]. In AD mouse models, reduced A-type Kv4 currents were associated with increased dendritic excitability and firing frequency of hippocampal neurons [26,59].

Voltage-clamp recordings of subicular neurons revealed significant increase in A-type K^+^ currents in AD mice compared to WT. Our finding differs from previous reports [26,59] possibly because of the differences in recorded cell types, studied mouse model, or disease stage. UCP4 overexpression in 3xTG AD mice had a mild, but not significant, effect on A-type K^+^ current density, nevertheless canceling the difference between WT and 3xTG UCP4 mice.

Interestingly, UCP4 overexpression had an opposite effect on A-type K^+^ current density in WT mice. In parallel, we assessed the effect of UCP4 overexpression on somatic and dendritic activity-related Ca^2+^ influx using combined 2-photon Ca^2+^ imaging and whole-cell patch-clamp, and found decrease in somatic activity-related Ca^2+^ influx in 3xTG AD mice compared to WT. This decrease in somatic activity-related Ca^2+^ influx was reversed by UCP4 overexpression in 3xTG AD mice, bringing Ca^2+^ transient levels back to control values. Overall, UCP4 overexpression in 3xTG AD mice enhanced neuronal excitability, likely due to reduction in A-type K^+^ current density, and increased the activity-related Ca^2+^ influx. Neither neuronal firing frequency or activity-related Ca^2+^ influx was significantly affected by UCP4 in WT mice. UCP4 overexpression had weaker - and in some cases opposite - effects on ion channel activity and neuronal excitability in aged WT mice compared with 3xTG-AD mice. These results suggest that astrocytic mitochondrial UCP4 differentially modulates K^+^ and Ca^2+^ conductances in subicular neurons in healthy aging versus disease.

Our next step was to identify the mechanisms linking astrocytic UCP4 with changes in neuronal K^+^ and Ca^2+^ conductances. Interestingly, similar modulation of K^+^ and Ca^2+^ currents were previously reported when GDNF level was increased in cultured midbrain neurons, with GDNF bath application causing an increase in cellular excitability by reducing A-type K^+^ currents and a parallel facilitation of Ca^2+^ influx through high voltage-gated Ca^2+^ channels [46]. Building on the similarities between GDNF- and UCP4-mediated effects on neuronal function and ion channels, we reasoned that overexpression of UCP4 in astrocytes could upregulate levels of neurotrophic factors produced by astrocytes such as GDNF. Emerging evidence points to a crucial role of GDNF in neuronal survival and plasticity [60]. Multiple studies have reported de novo expression of the GDNF by glial cells, including astrocytes, in the injured brain promoting neuronal survival and repair [61,62,63,64,65,66,67]. Mice heterozygous for GDNF deletion were reported to have impaired spatial learning, highlighting the importance of GDNF for hippocampal function [68]. GDNF expression was shown to decline with aging and is further altered in AD mouse models and human patients [69,70,71,72,73,74,75,76]. Conversely, studies on AD murine models have reported beneficial effects of increased GDNF expression [77,78], such as improved spatial learning and memory in 3xTG AD mice [78].

To investigate a potential link between UCP4 and GDNF, we quantified GDNF level in astrocytes overexpressing UCP4 and found a 1.3-fold increase in GDNF in astrocytes treated with UCP4. Furthermore, acute bath application of GDNF in slices increased excitability and activity-related Ca^2+^ influx in 3xTG AD mice subicular neurons. Thus, GDNF action in neurons of 3xTG AD mice closely resembled effects of UCP4 overexpression. Interestingly, GDNF application did not have a significant effect on neuronal excitability and activity-related Ca^2+^ influx in aged WT mice, as was the case with UCP4 treatment.

In conclusion, this study presents evidence that overexpressing UCP4 in mitochondria of astrocytes of AD mice is not only able to prevent the buildup of the disease-related phenotype of AD mice when administered at pre-symptomatic stage, as shown previously [3], but can also positively impact the neuronal function of mice with established AD symptoms. Upregulation of UCP4 in astrocytes partially rescued A-type K^+^ and Cav channels function which may contribute to the observed enhancement of neuronal excitability and improved network activity in AD mice. Finally, we propose that the upregulation of GDNF or other neurotrophic factors constitutes a plausible link between astrocyte mitochondria and the maintenance of neuronal function in the context of AD-related neurodegeneration.

## Data Availability

Data are available upon request. MATLAB codes are stored in GitHub repository https://github.com/GaifullinaAisylu/SWR-Average-Spectrograms (accessed on 16 January 2026).

## Supplementary Materials

The following supporting information can be downloaded at: https://www.mdpi.com/article/10.3390/cells15070597/s1, Supplementary Methods, Figures and Tables.

## Abbreviations

The following abbreviations are used in this manuscript:

ACSF	Artificial cerebrospinal fluid
AD	Alzheimer’s disease
Cav	Voltage-gated Ca^2+^ channel
DMEM	Dulbecco’s Modified Eagle Medium
DVC	Digital ventilated cage
GDNF	Glial derived neurotrophic factor
GFAP	Glial fibrillary acidic protein (GFAP)
LFP	Local field potentials
ROI	Region of interest
SWR	Sharp wave ripple
UCP4	Uncoupling protein 4
