# Supplementary material for: Astrocyte Mitochondrial UCP4 Reprograms Neuronal Network Oscillations via GDNF-Dependent K+-Ca2+ Signaling in Alzheimer’s Disease Mice
Source: Cells. 2026 Mar 27;15(7):597. doi: 10.3390/cells15070597 (PMC13072395; doi:10.3390/cells15070597)
Supplement: Supplementary file 1 [file cells-15-00597-s001.zip › cells-4192521-supplementary.pdf]

Astrocyte mitochondrial UCP4 reprograms neuronal network oscillations via GDNF dependent K^+-Ca^{2+} signaling in Alzheimer's disease mice.

Aisylu Gaifullina, Chaima Belhi, Leonardo Restivo, and Jean-Yves Chatton

Supplementary Methods

Whole-cell voltage-clamp Ca^{2+} recordings

Whole-cell voltage-clamp Ca^{2+} (Cav) measurements were executed using the recording ACSF supplemented with in mM: 5 CsCl, 15 TEA, 2.5 4-AP, 600 nM TTX, 20 μM CNQX, 4 μM SR 95531 and 10 μM DL-AP5 with pH of 7.4 and osmolarity of 300-310mOsm/Kg. Patch pipettes (2.5-3.5 MΩ) for Cav channel recordings were filled with internal solution containing in mM: 180 N-Methyl-D-glucamine, 40 HEPES, 0.1 EGTA, 4 MgCl2, 5 Na-ATP, 1 Lithium-GTP, 0.1% neurobiotin tracer, with pH of 7.35 adjusted with H_2SO_4.

To assess the steady-state inactivation of low-voltage activated Ca^{2+} currents, neurons were held at -50mV and then pre-pulsed to -100mV for 1.5sec followed by application of series of 1.5sec hyperpolarizing pulses from -120mV to -50mV with 10mV increment. To measure the steady-state activation of low-voltage activated Ca^{2+} currents, neurons were hold at -50mV and then pre-pulsed to -100mV for 1.5sec, afterwards a series of 1.5sec hyperpolarizing pulses from -100mV to -50mV with 10mV increment were applied to evoke Ca^{2+} currents. To calculate the voltage at half inactivation (V50 inactivation) and slope of inactivation, data were fitted with Boltzmann function in GraphPad Prism:

$$G = (1\text{-Gmax})/(1+\exp((\text{V50 inactivation-V})/\text{kpinactivation}))+\text{Gmax}$$

where Gmax is the maximal conductance, V is the respective test potential, V50 inactivation is the half-maximal inactivation voltage and k inactivation is the inactivation slope factor. The slope factor (*k*) is reported as a positive value.

To calculate the voltage at half activation (V50 activation) and slope of activation, data were fitted with Boltzmann function in GraphPad Prism:

$$G = \text{Gmax}/(1+\exp((\text{V50 activation-V})/\text{kactivation}))$$

where Gmax is the maximal conductance, V is the respective test potential, V50 activation is the half-maximal activation voltage and k activation is the activation slope factor. The slope factor (*k*) is reported as a positive value.

Neurons were voltage-clamped and recorded before and after application of Z944 (4μM) a T-type Ca^{2+} current blocker, to prove the molecular identity of a low-voltage activated Ca^{2+} currents. Pharmacological measurements were carried out using steady-state activation protocol. T-type low-voltage activated Ca^{2+} current density and inactivation time constant were calculated from the trace recorded at −100 mV. Current-clamp recordings were

performed before and after 10min application of Z944 (4μM) to show T-type Ca^{2+} channel mediated origin of burst activity. To calculate the contribution of T-type Ca^{2+} channels into overall somatic and dendritic Ca^{2+} transients, neuronal spike activity and Ca^{2+} transients before and after 10min application of Z944 (4μM). To study the effect of ryanodine receptors activation on neuronal firing and Ca^{2+} transients, cells were recorded before and after 10min application of caffeine (5mM).Membrane capacitance (Cm, pF), membrane resistance (Rm, MOhm), and membrane time constant (tau, ms) were obtained from the membrane test function in Clampfit during whole-cell voltage-clamp recordings. Input resistance was calculated during whole-cell current-clamp recordings using the following formula:

$$Rinput = (V2 - V1)/ \text{ injected test current},$$

where V2 is the voltage after test current injection and V1 is the voltage before test current injection. The injected test current used for these calculations was 200 pA.

Supplementary Figures

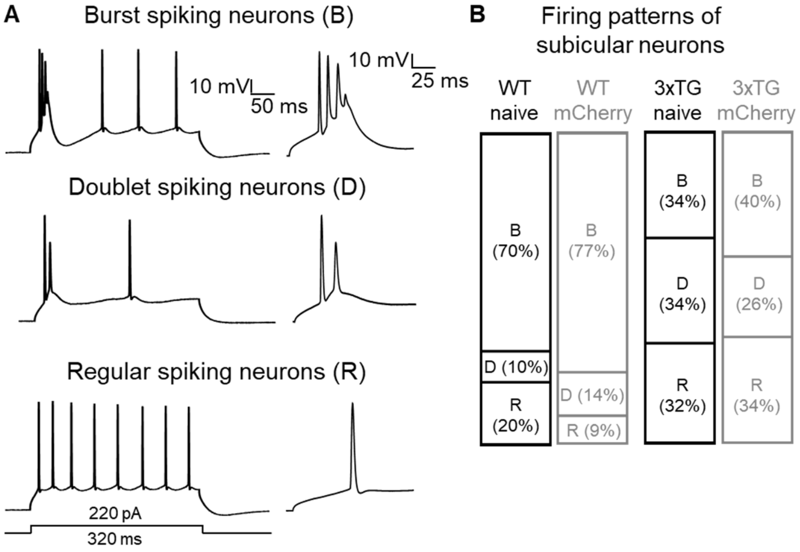

Figure S1. Different firing patterns of subicular neurons**. (A)** Representative traces from burst, doublet and regular spiking subicular neurons. Action potentials were elicited by 220pA current injection. **(B)** Proportions of firing patterns of subicular neurons for WT, WT mCherry, 3xTG AD, and 3xTG mCherry. No significant difference was found between naïve and mCherry injected WT and 3xTG AD mice. Statistics according to Chis-quare test. Additional details of the statistical comparisons are provided in Table S7. WT naïve n neurons=86, N mice=24; WT mCherry n neurons=49, N mice=5; 3xTG naïve n neurons=87, N mice=32; 3xTG mCherry n neurons=15, N mice=6.

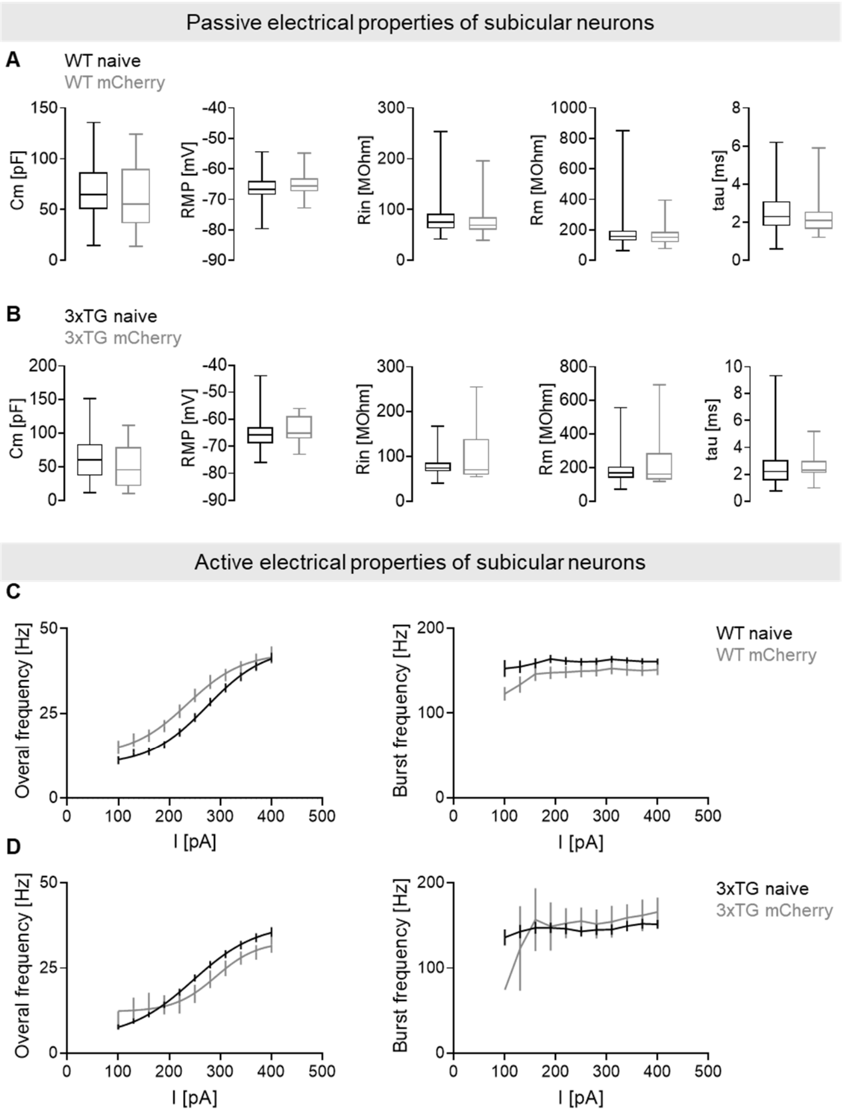

Figure S2. No difference in passive and active electrical properties between naïve and mCherry injected WT and 3xTG AD mice. **(A, B)** Comparisons of neuronal passive properties measured from naïve and mCherry expressing WT **(A)** and 3xTG AD **(B)** mice. Box and whiskers (min to max) plots depict membrane capacitance (Cm, pF), resting membrane potential (RMP, mV), input resistance (Rin, MOhm), membrane resistance (Rm, MOhm), and membrane time constant (tau, ms). No significant differences were found between naïve and mCherry mice (Mann-Whitney test). WT naïve n neurons=86, N mice=24; WT mCherry n neurons=49, N mice=5; 3xTG naïve n neurons=87, N mice=32; 3xTG mCherry n neurons=15, N mice=6. **(C, D)** Comparisons of neuronal active properties measured from naïve and mCherry expressing WT **(C)** and 3xTG AD **(D)** mice. Graphs depict the frequency (Hz) of overall evoked action potential firing (*left*) and frequency within the initial burst (*right*). Statistics according to multiple Mann-Whitney tests with false discovery rate correction; q values are reported. Additional details of the statistical comparisons are provided in Table S8. WT naïve n neurons=86, N mice=24; WT mCherry n neurons=49, N mice=5; 3xTG naïve n neurons=87, N mice=32; 3xTG mCherry n neurons=15, N mice=6. Data are presented as mean±SEM.

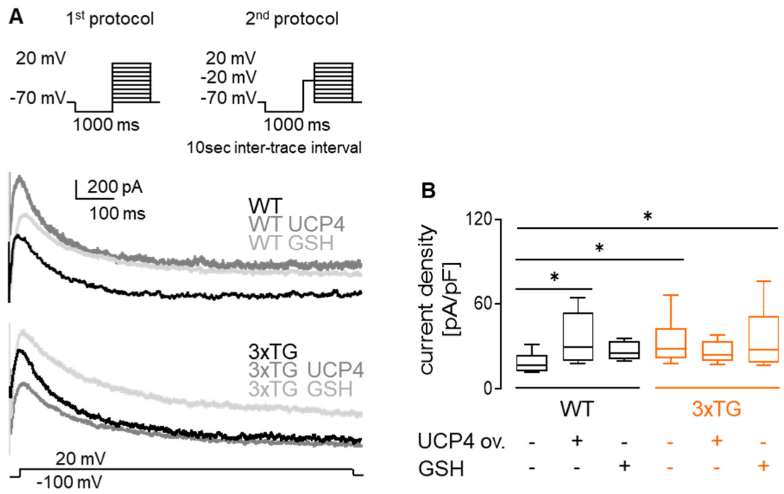

Figure S3. A-type K^+ currents in subicular neurons from WT and 3xTG AD mice. (**A**) Graphical description of the voltage-clamp protocol used to record A-type K^+ currents (*top*). Representative traces of A-type K^+ currents (pA) in subicular neurons from WT and 3xTG AD mice at +20mV step (*bottom*). (**B**) Box and whiskers plot comparing A-type K^+ currents density (pA/pF) evoked by +20mV voltage step in WT, WT UCP4, 3xTG, 3xTG UCP4, and the effect of 10mM glutathione (GSH) intracellular dialysis (*right*). UCP4 overexpression increased A-type K^+ currents density in WT mice, while intracellular dialysis of 10mM glutathione had no effect on A-type K^+ currents. UCP4 exerted a genotype-dependent effect on A-type K^+ current density, shifting values in 3xTG AD mice toward WT, although the direct comparison between 3xTG and 3xTG UCP4 did not reach statistical significance. The latest suggests that the observed changes in A-type K^+ currents are unlikely to result from alterations in cellular redox status in 3xTG AD mice. 2-way ANOVA revealed no significant effects of the genotype-treatment interaction ($F_{(2, 35)}$=2.657, p=0.0843), genotype ($F_{(1, 35)}$=0.8902, p=0.3519) or treatment ($F_{(2, 35)}$=0.5200, p=0.5991). *p<0.05 (by Fisher's LSD post-hoc test). Additional details of the statistical comparisons are provided in Table S9. WT n neurons=8, N mice=3; WT UCP4 n neurons=8, N mice=2; WT glutathione n neurons=4, N mice=2 mice; 3xTG n neurons=9, N mice=4; 3xTG UCP4 n neurons=6, N mice=2; 3xTG glutathione n neurons=6, N mice=3.

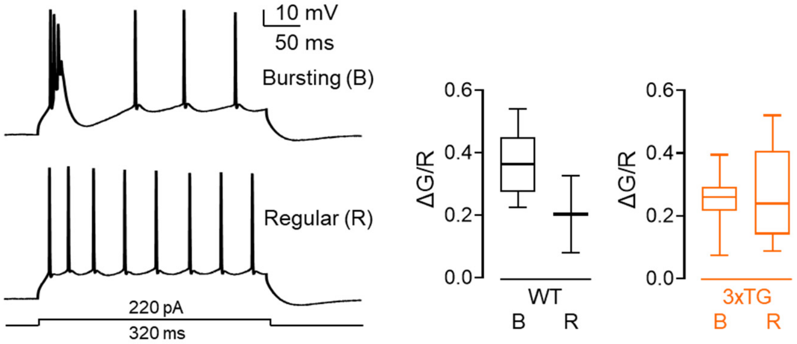

Figure S4. No difference in Ca^{2+} transient amplitude between bursting and regular spiking neurons. (*Left*) Original current-clamp traces from bursting and regular subicular neurons triggered by injection of the 220pA current from WT mice. (*Right*) Comparisons of somatic Ca^{2+} transients from bursting and regular spiking neurons recorded in ACSF from WT (B n neurons=18, N mice=10; R n neurons=2, N mice=2) and 3xTG AD mice (B n neurons=14, N mice=17; R n neurons=15, N mice=12). Statistics according to Mann-Whitney test. Additional details of the statistical comparisons are provided in Table S10. Data are presented as box and whiskers (min to max) plots.

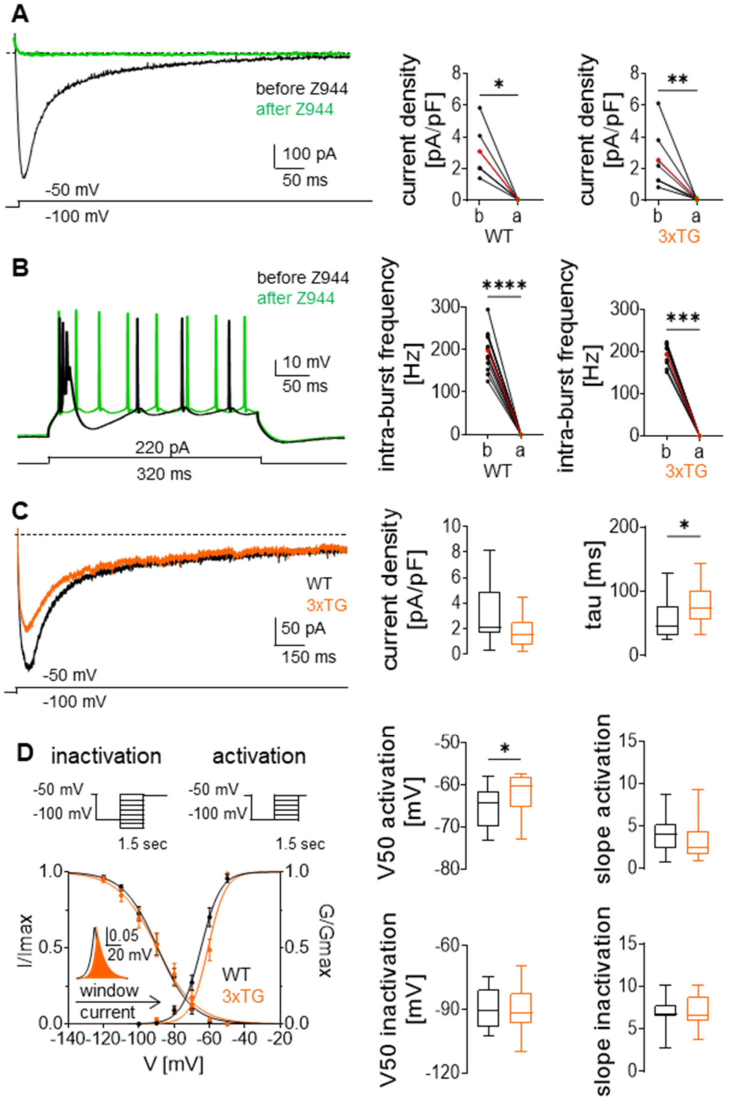

Figure S5. Low-voltage activated Ca^{2+} currents in subicular neurons from WT and 3xTG AD mice. (**A**) Representative traces of Ca^{2+} currents (pA) prepulsed to -100mV for 1.5sec from holding voltage of 50mV and then stepped to 50mV for 1.5 seconds before (black line) and after (green line) bath application of the T-type Ca^{2+} channel blocker 4µM Z944 (*left*). T-type Ca^{2+} channel density (pA/pF; before after plots) in subicular neurons before and after Z944 application (*right*). *p<0.05, **p<0.01 (Wilcoxon test). WT n neurons=7, N mice=3; 3xTG n neurons=5, N mice=3. Mean values are indicated in red. (**B**). Representative current-clamp traces of action potentials evoked by 220pA current injection for 320ms before (black line) and after (green line) 10min bath application of T-type Ca^{2+} channel blocker 4µM Z944 (*left*). Intra-burst frequency (Hz) in subicular neurons before and after Z944 application (*right*). ***p<0.001, ****p<0.0001 (Wilcoxon test). WT n neurons=14, N mice=8; 3xTG n neurons=10, N mice=5. Mean values are indicated in red. (**C**). Representative low voltage activated T-type Ca^{2+} currents from WT (black line) and 3xTG AD (orange line) mice (*left*). T-type Ca^{2+} current density (pA/pF) and inactivation time constant (ms) at 100mV (*right*). *p<0.05 (Mann-Whitney test). WT n neurons=17, N mice=7; 3xTG n neurons=17, N mice=6. The T-type Ca^{2+} current amplitude was significantly reduced in 3xTG AD compared to WT mice (WT 274±41.2pA; 3xTG 140±17.5pA, p=0.0108 (Mann-Whitney test), but did not reach significance when normalized to membrane capacitance p=0.0851 (Mann-Whitney test). (**D**) Steady state voltage dependent inactivation and activation kinetics of low voltage activated T-type

Ca^{2+} currents from subicular neurons of WT (black) and 3xTG AD (orange) mice (*left*). Inactivation and activation protocols are shown as insets. Inactivation and activation currents (I /Imax and G/Gmax respectively) are depicted along with the window current (overlap of the two curves, shown in the zoomed up inset). Data are presented as mean±SEM and fitted with Boltzmann function in GraphPad Prism. Half activation (V50 activation) and inactivation (V50 inactivation) voltage (mV), slope of activation and inactivation of T-type Ca^{2+} channels (*right*) presented as box and whiskers plots. *$p<0.05$ (Mann-Whitney test). WT n neurons=17, N mice=7; 3xTG n neurons=17, N mice=6. Additional details of the statistical comparisons are provided in Table S11.

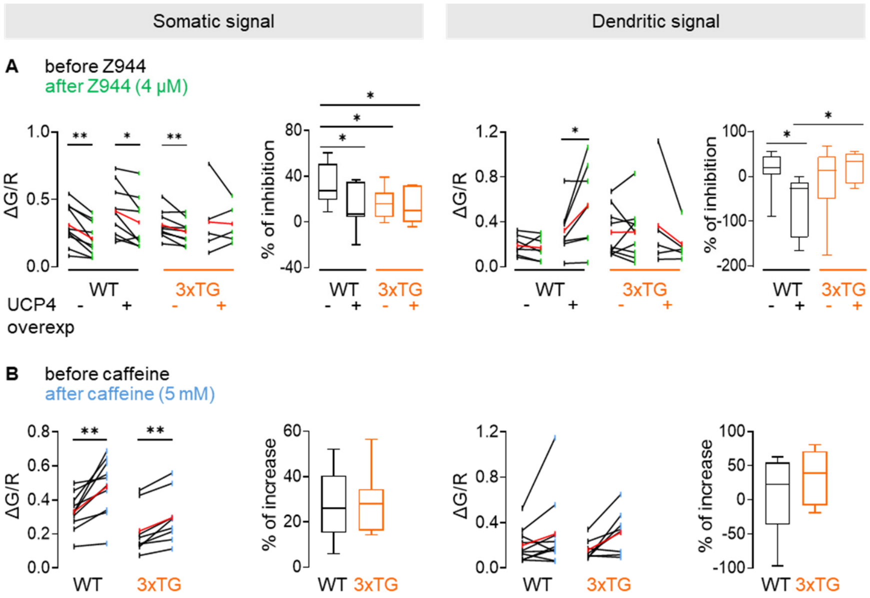

Figure S6. Effects of T-type Ca^{2+} channel inhibition and ryanodine receptor activation on somatic and dendritic Ca^{2+} transients. (**A**) Ca^{2+} transients measured in soma (*left graphs*) and dendrites (*right graphs*) of subicular neurons evoked by injection of 220pA from WT and 3xTG AD mice subjected to UCP4 treatment or not, before and after 4µM Z944 bath application for 10min. The amplitude of Ca^{2+} transients (in ΔG/R; before-after plots) for individual cells is shown along with comparison of the Z944-sensitive Ca^{2+} response (in percent; box and whiskers plots) between the two genotypes. Statistical significance was tested by Wilcoxon test (before-after plots) and 2-way ANOVA followed by Fisher's LSD multiple comparisons (box and whiskers plots) where *p<0.05, **p<0.01. 2-way ANOVA analyses on Z944-sensitive somatic current revealed no significant effects of the genotype-treatment interaction ($F_{(1, 27)}$=2.344, p=0.1374), genotype ($F_{(1, 27)}$=1.742, p=0.198) or treatment ($F_{(1, 27)}$=2.989, p=0.0952). In contrast, 2-way ANOVA analyses on Z944-sensitive dendritic current revealed significant effects of the genotype-treatment interaction ($F_{(1, 24)}$=5.328, p=0.0299), but not of genotype ($F_{(1, 24)}$=1.945, p=0.1759) and treatment ($F_{(1, 24)}$=1.041, p=0.3177). Additional details of the statistical comparisons are provided in Table S12. WT somatic/dendritic ΔG/R n neurons=10/7, N mice=4/4; WT UCP4 somatic/dendritic ΔG/R n neurons=9/9, N mice=4/4; 3xTG somatic/dendritic ΔG/R n neurons=9/9, N mice=7/7; 3xTG UCP4 somatic/dendritic ΔG/R n neurons=5/5, N mice=4/4. Mean values are indicated in red. (**B**) Ca^{2+} transients measured in soma (*left graphs*) and dendrites (*right graphs*) of subicular neurons evoked by injection of 220pA from WT and 3xTG AD mice before and after 5mM caffeine bath application for 10min. The amplitude of Ca^{2+} transients (in ΔG/R; before-after plots) for individual cells is shown along with comparison of Ca^{2+} response enhancement (in percent; box and whiskers plots) between the two genotypes. Statistical difference according to Wilcoxon test (before-after plots) and Mann-Whitney test (box and whiskers plots) where **p<0.01. Additional details of the statistical comparisons are provided in Table S12. WT somatic/dendritic ΔG/R n neurons=10/10, N mice=8/8. 3xTG somatic/dendritic ΔG/R n neurons=8/8, N mice=6/6. Mean values are indicated in red.

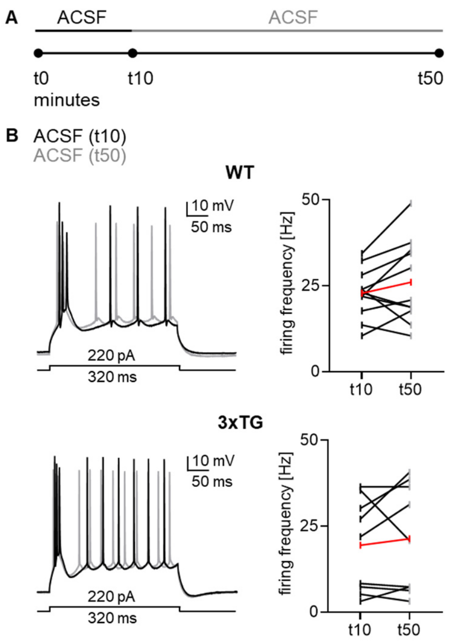

Figure S7. No significant change in neuronal firing frequency observed after 40min in whole-cell configuration. (**A**) Experimental design of long term whole-cell- current-clamp measurements in subicular neurons. In this control experiment, ACSF was kept throughout the recordings. The indicated times (t0, t10, and t50, in minutes) correspond to the ones used for GDNF experiments (Fig. 7A-B). (**B**) Action potentials were triggered by series of current injections (from -230pA to 400pA, 30pA increments, lasting each 320ms) in neurons of WT (*top graphs*) and in 3xTG AD (*bottom graphs*) mice. Original traces (*left*) at t10 (black) and t50 (grey) are depicted for both genotypes. Comparison of firing frequency at t10 and t50 (*right*) at 220pA current trace is shown for each recorded cell. No significant differences were found between the two time points (Wilcoxon test). Additional details of the statistical comparisons are provided in Table S13. WT n neurons=11, N mice=4; 3xTG n neurons=9, N mice=4. Mean values are indicated in red. Data are presented as before-after plots.

Supplementary Tables

Table S1. Home-cage behavioral analyses of locomotor activity and front/rear choice test (refers to Figure 2).

Locomotor activity

		Median	Minimum	Maximum	n/N	Mann-Whitney test
WT	ratio	0.76	0.49	0.93	21/9	*0.0496
3xTG	ratio	0.93	0.76	0.96	14/6	

Front/rear choice

	half rear			half front					
	Median	Minimum	Maximum	Median	Minimum	Maximum	n/N	Mann-Whitney test	Chi^2 test
WT	0.07	0.03	0.12	0.06	0.04	0.1	21/9	0.8182	*0.0300
3xTG	0.07	0.04	0.09	0.04	0.02	0.05	14/6	**0.0087	

Ratio of light/dark phase activity of WT and 3xTG AD mice. Front/rear cage occupancy of WT and 3xTG AD mice. n indicates the number of mice, N represents the number of cages. Statistical differences according to Mann-Whitney test and Chi-square test.

Table S2. Occurrence and duration of CA1 and Subicular (Sub) SWRs. Firing frequency of single-cell subicular neurons (refers to Figure 3).

SWR CA1 occurrence

			Median	Minimum	Maximum	n/N
WT	UCP4 -	CA1 occurrence (Hz)	1.9	0.05	7.97	14/5
	UCP4 +	CA1 occurrence (Hz)	0.71	0.02	5.22	24/5
3xTG	UCP4 -	CA1 occurrence (Hz)	3.26	1.31	7.7	15/6
	UCP4 +	CA1 occurrence (Hz)	1.82	0.58	6.13	12/3

SWR Sub occurrence

			Median	Minimum	Maximum	n/N
WT	UCP4 -	Sub occurrence (Hz)	1.5	0.05	6.05	14/5
	UCP4 +	Sub occurrence (Hz)	2.12	0.24	4.8	23/5
3xTG	UCP4 -	Sub occurrence (Hz)	3.36	2.1	4.12	12/5
	UCP4 +	Sub occurrence (Hz)	1.92	0.18	4.13	12/3

SWR CA1 duration

			Median	Minimum	Maximum	n/N
WT	UCP4 -	CA1 duration (ms)	49.65	48.64	66.05	11/5
	UCP4 +	CA1 duration (ms)	48.22	44.32	51.33	24/5
3xTG	UCP4 -	CA1 duration (ms)	49.04	46.82	53.32	15/6
	UCP4 +	CA1 duration (ms)	49.2	46.94	65.39	12/3

SWR Sub duration

			Median	Minimum	Maximum	n/N
WT	UCP4 -	Sub duration (ms)	46.76	38.89	65.83	14/5
	UCP4 +	Sub duration (ms)	49.88	44.49	67.4	23/5
3xTG	UCP4 -	Sub duration (ms)	49.62	48.86	50.72	12/5
	UCP4 +	Sub duration (ms)	48.14	45.47	57.38	12/3

Neuronal firing frequency

			Median	Minimum	Maximum	n/N
WT	UCP4 -	Firing frequency (Hz)	18.8	3.13	67.7	135/29
	UCP4 +	Firing frequency (Hz)	18.25	3.13	51	28/7
3xTG	UCP4 -	Firing frequency (Hz)	15.6	3.13	40.6	102/38
	UCP4 +	Firing frequency (Hz)	22.4	7.29	50	26/6

2-way ANOVA (CA1 occurrence)

	F (DFn, DFd)	p value
Interaction	F (1, 61) = 0.7295	0.3964
Genotype	F (1, 61) = 4.723	*0.0336
Treatment	F (1, 61) = 4.808	*0.0321

2-way ANOVA (Sub occurrence)

	F (DFn, DFd)	p value
Interaction	F (1, 57) = 5.888	*0.0184
Genotype	F (1, 57) = 2.988	0.0893
Treatment	F (1, 57) = 0.4930	0.4854

2-way ANOVA (CA1 duration)

	F (DFn, DFd)	p value
Interaction	F (1, 58) = 11.03	**0.0016
Genotype	F (1, 58) = 0.1025	0.75
Treatment	F (1, 58) = 0.3543	0.554

2-way ANOVA (Sub duration)

	F (DFn, DFd)	p value
Interaction	F (1, 57) = 2.377	0.1287
Genotype	F (1, 57) = 0.005113	0.9432
Treatment	F (1, 57) = 0.6606	0.4197

2-way ANOVA (firing frequency)

	F (DFn, DFd)	p value
Interaction	F (1, 287) = 4.589	*0.0330
Genotype	F (1, 287) = 0.09115	0.7629
Treatment	F (1, 287) = 0.6279	0.4288

Uncorrected Fisher's LSD tests (CA1 occurrence)

WT UCP4± vs 3xTG UCP4±			p value
WT UCP4 -	vs	WT UCP4 +	0.3113
WT UCP4 -	vs	3xTG UCP4 -	*0.0409
WT UCP4 -	vs	3xTG UCP4 +	0.9899
WT UCP4 +	vs	3xTG UCP4 -	**0.0012
WT UCP4 +	vs	3xTG UCP4 +	0.3424
3xTG UCP4 -	vs	3xTG UCP4 +	*0.048

Uncorrected Fisher's LSD tests (Sub occurrence)

WT UCP4± vs 3xTG UCP4±			p value
WT UCP4 -	vs	WT UCP4 +	0.1824
WT UCP4 -	vs	3xTG UCP4 -	**0.0069
WT UCP4 -	vs	3xTG UCP4 +	0.4916
WT UCP4 +	vs	3xTG UCP4 -	0.0754
WT UCP4 +	vs	3xTG UCP4 +	0.605
3xTG UCP4 -	vs	3xTG UCP4 +	*0.0467

Uncorrected Fisher's LSD tests (CA1 duration)

WT UCP4± vs 3xTG UCP4±			p value
WT UCP4 -	vs	WT UCP4 +	**0.0059
WT UCP4 -	vs	3xTG UCP4 -	*0.0492
WT UCP4 -	vs	3xTG UCP4 +	0.8616
WT UCP4 +	vs	3xTG UCP4 -	0.4628
WT UCP4 +	vs	3xTG UCP4 +	**0.0082
3xTG UCP4 -	vs	3xTG UCP4 +	0.0665

Uncorrected Fisher's LSD tests (Sub duration)

WT UCP4± vs 3xTG UCP4±			p value
WT UCP4 -	vs	WT UCP4 +	0.0706
WT UCP4 -	vs	3xTG UCP4 -	0.2812
WT UCP4 -	vs	3xTG UCP4 +	0.5533
WT UCP4 +	vs	3xTG UCP4 -	0.5829
WT UCP4 +	vs	3xTG UCP4 +	0.278
3xTG UCP4 -	vs	3xTG UCP4 +	0.6375

Uncorrected Fisher's LSD tests (firing frequency)

WT UCP4± vs 3xTG UCP4±			p value
WT UCP4 -	vs	WT UCP4 +	0.3267
WT UCP4 -	vs	3xTG UCP4 -	**0.0052
WT UCP4 -	vs	3xTG UCP4 +	0.7295
WT UCP4 +	vs	3xTG UCP4 -	0.4388
WT UCP4 +	vs	3xTG UCP4 +	0.308
3xTG UCP4 -	vs	3xTG UCP4 +	*0.0444

Characterization of SWR parameters and neuronal firing in WT, WT overexpressing UCP4, 3xTG and 3xTG overexpressing UCP4 mice. n indicates the number of recorded brain slices, N represents the number of

recorded mice. For analyses of neuronal firing frequency, n stands for the number of recorded neurons, N represents the number of recorded mice. Statistical differences according to 2-way ANOVA followed by Fishers LSD multiple comparisons test.

Table S3. Effect of UCP4 overexpression on A-type potassium currents (refers to Figure 4).

A-type potassium current density

			Median	Minimum	Maximum	n/N
WT	UCP4 -	current density (pA/pF)	16.79	11.73	31.16	8/3
	UCP4 +	current density (pA/pF)	29.43	17.7	64.01	8/2
3xTG	UCP4 -	current density (pA/pF)	28	18	66	9/4
	UCP4 +	current density (pA/pF)	23.62	17.09	38.07	6/2

2-way ANOVA

	F (DFn, DFd)	p value
Interaction	F (1, 27) = 6.016	*0.0209
Genotype	F (1, 27) = 0.2456	0.6242
Treatment	F (1, 27) = 0.9286	0.3438

Uncorrected Fisher's LSD tests

WT UCP4± vs 3xTG UCP4±			p value
WT UCP4 -	vs	WT UCP4 +	*0.0196
WT UCP4 -	vs	3xTG UCP4 -	*0.0362
WT UCP4 -	vs	3xTG UCP4 +	0.3351
WT UCP4 +	vs	3xTG UCP4 -	0.7291
WT UCP4 +	vs	3xTG UCP4 +	0.1991
3xTG UCP4 -	vs	3xTG UCP4 +	0.3139

A-type potassium currents in subicular neurons of WT, WT overexpressing UCP4, 3xTG and 3xTG overexpressing UCP4 mice. n indicates the number of recorded neurons, N represents the number of recorded mice. Statistical differences according to 2-way ANOVA followed by Fishers LSD multiple comparisons test.

Table S4. UCP4-dependent modulation of somatic and dendritic neuronal activity-associated Ca^{2+} influx (refers to Figure 5).

Neuronal activity-associated Ca^{2+} influx

			somatic Ca^{2+} influx				dendritic Ca^{2+} influx			
			Median	Minimum	Maximum	n/N	Median	Minimum	Maximum	n/N
WT	UCP4 -	ΔG/R	0.35	0.23	0.54	20/12	0.2	0.07	0.81	18/12
	UCP4 +	ΔG/R	0.39	0.15	0.73	17/5	0.25	0.03	0.76	17/5
3xTG	UCP4 -	ΔG/R	0.25	0.07	0.52	29/14	0.14	0.07	0.67	27/15
	UCP4 +	ΔG/R	0.35	0.1	0.76	14/4	0.26	0.06	1.12	12/4

2-way ANOVA (somatic Ca^{2+} influx)

	F (DFn, DFd)	p value
Interaction	F (1, 76) = 0.6349	0.428
Genotype	F (1, 76) = 4.716	*0.033
Treatment	F (1, 76) = 5.100	*0.0268

2-way ANOVA (dendritic Ca^{2+} influx)

	F (DFn, DFd)	p value
Interaction	F (1, 70) = 0.7452	0.3909
Genotype	F (1, 70) = 0.03710	0.8478
Treatment	F (1, 70) = 3.090	0.0831

Uncorrected Fisher's LSD tests (somatic Ca^{2+} influx)

WT UCP4± vs 3xTG UCP4±			p value
WT UCP4 -	vs	WT UCP4 +	0.3079
WT UCP4 -	vs	3xTG UCP4 -	*0.0205
WT UCP4 -	vs	3xTG UCP4 +	0.9542
WT UCP4 +	vs	3xTG UCP4 -	**0.0012
WT UCP4 +	vs	3xTG UCP4 +	0.3803
3xTG UCP4 -	vs	3xTG UCP4 +	*0.0327

Uncorrected Fisher's LSD tests (dendritic Ca^{2+} influx)

WT UCP4± vs 3xTG UCP4±			p value
WT UCP4 -	vs	WT UCP4 +	0.5238
WT UCP4 -	vs	3xTG UCP4 -	0.4034
WT UCP4 -	vs	3xTG UCP4 +	0.3124
WT UCP4 +	vs	3xTG UCP4 -	0.1315
WT UCP4 +	vs	3xTG UCP4 +	0.6679
3xTG UCP4 -	vs	3xTG UCP4 +	0.0715

Somatic and dendritic neuronal activity-associated Ca^{2+} influx in subicular neurons of WT, WT overexpressing UCP4, 3xTG and 3xTG overexpressing UCP4 mice. n indicates the number of recorded neurons, N represents the number of recorded mice. Statistical differences according to 2-way ANOVA followed by Fishers LSD multiple comparisons test.

Table S5. Correlation of UCP4 and GDNF expression (refers to Figure 6).

		GDNF level				
		Median	Minimum	Maximum	n/N	Mann-Whitney test
WT	GDNF -	0.18	0.13	0.21	6/6	*0.0221
	GDNF +	0.25	0.17	0.28	7/7	

GDNF expression in astrocytes of WT mice transduced with control mCherry and UCP4 viruses. n indicates the number of recorded neurons, N represents the number of recorded mice. Statistical differences according to Mann-Whitney test.

Table S6. Effect of GDNF on neuronal firing frequency and activity-related Ca^{2+} transients (refers to Figure 7).

Neuronal firing frequency

		before 2 nM GDNF			after 2 nM GDNF				
		Median	Minimum	Maximum	Median	Minimum	Maximum	n/N	Wilcoxon test
WT	Firing frequency (Hz)	15.1	8.3	29.2	15.1	9.4	33.3	8/5	0.2969
3xTG	Firing frequency (Hz)	14.6	6.3	17.7	18.8	12.5	38.5	7/4	*0.0313

Neuronal activity-associated Ca^{2+} influx

			somatic Ca^{2+} influx				dendritic Ca^{2+} influx			
			Median	Minimum	Maximum	n/N	Median	Minimum	Maximum	n/N
WT	GDNF -	ΔG/R	0.35	0.23	0.54	20/12	0.2	0.07	0.8	18/12
	GDNF +	ΔG/R	0.25	0.16	0.4	8/3	0.33	0.21	0.5	5/3
3xTG	GDNF -	ΔG/R	0.25	0.07	0.52	29/14	0.14	0.07	0.67	27/15
	GDNF +	ΔG/R	0.32	0.14	0.55	15/6	0.32	0.15	0.58	13/6

2-way ANOVA (somatic Ca^{2+} influx)

	F (DFn, DFd)	p value
Interaction	F (1, 68) = 7.660	**0.0073
Genotype	F (1, 68) = 0.4486	0.5053
Treatment	F (1, 68) = 0.002787	0.9581

2-way ANOVA (dendritic Ca^{2+} influx)

	F (DFn, DFd)	p value
Interaction	F (1, 59) = 0.8265	0.367
Genotype	F (1, 59) = 0.02269	0.8808
Treatment	F (1, 59) = 4.022	*0.0495

Uncorrected Fisher's LSD tests (somatic Ca^{2+} influx)

WT GDNF± vs 3xTG GDNF±			p value
WT GDNF -	vs	WT GDNF +	0.081
WT GDNF -	vs	3xTG GDNF -	**0.0028
WT GDNF -	vs	3xTG GDNF +	0.5801
WT GDNF +	vs	3xTG GDNF -	0.6861
WT GDNF +	vs	3xTG GDNF +	0.2123
3xTG GDNF -	vs	3xTG GDNF +	*0.0282

Uncorrected Fisher's LSD tests (dendritic Ca^{2+} influx)

WT GDNF± vs 3xTG GDNF±			p value
WT GDNF -	vs	WT GDNF +	0.5124
WT GDNF -	vs	3xTG GDNF -	0.2941
WT GDNF -	vs	3xTG GDNF +	0.1268
WT GDNF +	vs	3xTG GDNF -	0.1834
WT GDNF +	vs	3xTG GDNF +	0.6629
3xTG GDNF -	vs	3xTG GDNF +	*0.011

Neuronal firing frequency and activity-related Ca^{2+} transients in the absence and presence of the GDNF. n indicates the number of recorded neurons, N represents the number of recorded mice. Statistical differences according to 2-way ANOVA followed by Fishers LSD multiple comparisons test.

Table S7. Comparisons of the proportions of neuronal firing patterns in WT and 3xTG mice (refers to Figure S1).

Neuronal firing patterns						
	WT naive	WT mCherry	Chi^2 test	3xTG naive	3xTG mCherry	Chi^2 test
Burst spiking	70%	77%	0.0753	34%	40%	0.4462
Doublet spiking	10%	14%		34%	26%	
Regular spiking	20%	9%		32%	34%	
n/N	86/24	49/5		87/32	15/6	

The number of burst, doublet and regular spiking subicular neurons for WT naive, WT mCherry, 3xTG naive and 3xTG mCherry mice is given in percentage. n indicates the number of recorded neurons, N represents the number of recorded mice. Statistical differences according to Chi-square test.

Table S8. Comparisons of passive and active properties of WT naïve and WT mCherry; 3xTG naïve and 3xTG mCherry mice (refers to Figure S2).

Passive electrical properties of subicular neurons

		WT naïve				WT mCherry				Mann-Whitney test
		Median	Minimum	Maximum	n/N	Median	Minimum	Maximum	n/N	
WT	capacitance (pF)	64.74	14.51	135.9	86/24	55.42	13.81	124.1	49/5	0.1192
WT	RMP (mV)	-66.76	-79.56	-54.41	86/24	-65.58	-72.85	-54.81	49/5	0.0912
WT	Rin (Mohm)	74.9	42	253.6	86/24	69	39.63	196.1	49/5	0.0783
WT	Rm (Mohm)	157	63.7	849.5	86/24	152.2	78.2	395.5	49/5	0.2438
WT	tau (ms)	2.3	0.6	6.2	86/24	2.1	1.2	5.9	49/5	0.2453

		3xTG naïve				3xTG mCherry				Mann-Whitney test
		Median	Minimum	Maximum	n/N	Median	Minimum	Maximum	n/N	
3xTG	capacitance (pF)	60.61	11.75	151.1	87/32	45.44	10.37	111.4	15/6	0.229
3xTG	RMP (mV)	-65.81	-76.03	-44	87/32	-65.16	-73	-56.03	15/6	0.3923
3xTG	Rin (Mohm)	74.07	40.98	167.3	87/32	70.32	54.7	255	15/6	0.9199
3xTG	Rm (Mohm)	171.1	71.85	555.2	87/32	162.3	118.5	690	15/6	0.859
3xTG	tau (ms)	2.2	0.8	9.3	87/32	2.3	1	5.2	15/6	0.4749

Overall frequency (Hz)

current (pA)	WT naive				WT mCherry				Multiple Mann-Whitney test
	MEAN	SD	SEM	n/N	MEAN	SD	SEM	n/N	
100	11.1	5.4	1.2	86/24	14.9	8.9	1.9	49/5	0.4158
130	13.4	6.3	1.1	86/24	17.2	10.4	1.9	49/5	0.4158
160	13.7	8.6	1.1	86/24	18.5	11.6	1.8	49/5	0.078
190	15.7	9.9	1.1	86/24	21.3	12.6	1.8	49/5	0.0236
220	19.4	11.3	1.2	86/24	25.6	14.2	2	49/5	0.0236
250	23.8	11.8	1.3	86/24	30.3	13.7	1.9	49/5	0.0236
280	28.4	11.8	1.3	86/24	34.3	14.3	2	49/5	0.0269
310	32.6	12.3	1.3	86/24	36.4	13	1.8	49/5	0.1489
340	35.8	11.9	1.3	86/24	38.3	14.8	2.1	49/5	0.4158
370	38.8	12.2	1.3	86/24	40.3	16.3	2.3	49/5	0.6427
400	41.5	13.1	1.4	86/24	42.3	17.5	2.5	49/5	0.8955

Overall frequency (Hz)

current (pA)	3xTG naive				3xTG mCherry				Multiple Mann-Whitney test
	MEAN	SD	SEM	n/N	MEAN	SD	SEM	n/N	
100	7.7	3.9	0.7	87/32	9.6	5.3	2.6	15/6	0.6095
130	9.4	5.5	0.8	87/32	13.2	7.7	3.2	15/6	0.3376
160	11.3	6.7	0.8	87/32	14.7	8.6	3.1	15/6	0.3737

190	14.2	8.5	0.9	87/32	14.2	9.5	2.8	15/6	0.8305
220	17.9	9.5	1.1	87/32	14.3	10.5	2.7	15/6	0.3376
250	22.1	9.9	1.1	87/32	17.4	10.4	2.7	15/6	0.3376
280	25.9	10.3	1.1	87/32	21.7	9.9	2.6	15/6	0.3376
310	29.1	10.9	1.2	87/32	24.9	8.9	2.3	15/6	0.3376
340	31.7	11.5	1.2	87/32	27.9	7.6	1.9	15/6	0.3376
370	33.8	12.2	1.3	87/32	29.6	8.3	2.1	15/6	0.3376
400	35.6	13.4	1.4	87/32	31.9	9.5	2.5	15/6	0.4632

Burst frequency (Hz)

current (pA)	WT naive				WT mCherry				Multiple Mann-Whitney test
	MEAN	SD	SEM	n/N	MEAN	SD	SEM	n/N	
100	152.8	39.2	10.1	86/24	122.8	33.4	8.1	49/5	0.0683
130	154.7	36.5	7.3	86/24	133.6	44.7	9.5	49/5	0.0683
160	158.9	36.5	5.8	86/24	146.2	46.9	8.3	49/5	0.1437
190	163.9	34.6	4.8	86/24	147.6	45.4	7.4	49/5	0.1437
220	161.5	34.6	4.7	86/24	148.4	44.1	7.2	49/5	0.1437
250	160.6	35	4.5	86/24	149.6	45	7.3	49/5	0.1437
280	161.1	32.8	4.2	86/24	150.1	42.6	6.9	49/5	0.1437
310	163.5	30.2	3.9	86/24	152.6	42.6	6.9	49/5	0.1437
340	162.3	28.1	3.6	86/24	151.3	43.4	7	49/5	0.1437
370	161.1	29.4	3.7	86/24	150.3	39.8	6.5	49/5	0.1437
400	160.9	29.2	3.7	86/24	151.2	38.9	6.3	49/5	0.1437

Burst frequency (Hz)

current (pA)	3xTG naive				3xTG mCherry				Multiple Mann-Whitney test
	MEAN	SD	SEM	n/N	MEAN	SD	SEM	n/N	
100	136.1	34.1	9.5	87/32	74.6	0	0	15/6	
130	142.8	36.1	7.9	87/32	123.1	70.4	49.7	15/6	0.8136
160	147.2	36.8	7.1	87/32	156.9	63.9	36.8	15/6	0.8136
190	147.2	33.9	6.2	87/32	148.8	56.7	28.3	15/6	0.8654
220	146.2	34.6	6.3	87/32	152.6	43.5	17.7	15/6	0.8136
250	143.1	33.3	6.1	87/32	155.3	38.6	15.7	15/6	0.8136
280	144.9	34.1	6.2	87/32	151.7	41.7	17.1	15/6	0.8136
310	145.2	33.6	6.2	87/32	154.4	45.6	18.6	15/6	0.8136
340	149.4	32.4	5.9	87/32	159.2	38.7	15.8	15/6	0.8136
370	152.1	31.8	5.8	87/32	161.8	45.3	18.5	15/6	0.8136
400	151.4	28.4	5.3	87/32	165.9	41.7	17	15/6	0.8136

Membrane capacitance (pF), resting membrane potential (RMP, mV), input resistance (Rin, Mohm), membrane resistance (Rm, Mohm) and membrane time constant (tau, ms) of subicular neurons for WT naive, WT mCherry, 3xTG naive and 3xTG mCherry mice. n indicates the number of recorded neurons, N represents the number of recorded mice. Statistical differences according to Mann-Whitney test.

Overall and burst firing frequency of subicular neurons for WT naïve, WT mCherry, 3xTG naive and 3xTG mCherry mice at given current injection. n indicates the number of recorded neurons, N represents the number of recorded mice. Statistical differences according to Mann-Whitney test.

Table S9. The effect of UCP4 and GSH on A-type K^+ current density (refers to Figure S3).

A-type potassium current density

			Median	Minimum	Maximum	n/N
WT	UCP4 -	current density (pA/pF)	16.79	11.73	31.16	8/3
	UCP4 +	current density (pA/pF)	29.43	17.7	64.01	8/2
	GSH	current density (pA/pF)	25.37	19.57	35.53	4/2
3xTG	UCP4 -	current density (pA/pF)	28	18	66	9/4
	UCP4 +	current density (pA/pF)	23.62	17.09	38.07	6/2
	GSH	current density (pA/pF)	27.61	16.63	58.92	6/3

2-way ANOVA (A-type potassium current)

	F (DFn, DFd)	p value
Interaction	F (2, 35) = 2.657	0.0843
Genotype	F (1, 35) = 0.8902	0.3519
Treatment	F (2, 35) = 0.5200	0.5991

Uncorrected Fisher's LSD tests
(A-type potassium current)

WT UCP4± vs 3xTG UCP4±			p value
WT UCP4-	vs	WT UCP4+	*0.0283
WT UCP4-	vs	WT GSH	0.3921
WT UCP4-	vs	3xTG UCP4-	*0.0498
WT UCP4-	vs	3xTG UCP4+	0.3718
WT UCP4-	vs	3xTG GSH	*0.0463
WT UCP4+	vs	WT GSH	0.3235
WT UCP4+	vs	3xTG UCP4-	0.749
WT UCP4+	vs	3xTG UCP4+	0.2331
WT UCP4+	vs	3xTG GSH	0.9587
WT GSH	vs	3xTG UCP4-	0.4525
WT GSH	vs	3xTG UCP4+	0.9484
WT GSH	vs	3xTG GSH	0.3709
3xTG UCP4-	vs	3xTG UCP4+	0.3506
3xTG UCP4-	vs	3xTG GSH	0.8087
3xTG UCP4+	vs	3xTG GSH	0.2848

A-type K^+ current density in subicular neurons of WT, WT overexpressing UCP4, WT treated with 10 mM glutathione (GSH), 3xTG, 3xTG overexpressing UCP4 and 3xTG treated with 10 mM glutathione (GSH). n indicates the number of recorded neurons, N represents the number of recorded mice. Statistical differences according to 2-way ANOVA followed by Fishers LSD multiple comparisons test.

Table S10. Neuronal activity-associated Ca^{2+} influx in burst and regular spiking subicular neurons (refers to Figure S4).

			Neuronal activity-associated somatic Ca^{2+} influx				
			Median	Minimum	Maximum	n/N	Mann-Whitney test
WT	burst	ΔG/R	0.36	0.22	0.54	18/10	0.2381
	regular	ΔG/R	0.2	0.08	0.32	2/2	
3xTG	burst	ΔG/R	0.26	0.07	0.39	14/14	0.8805
	regular	ΔG/R	0.23	0.08	0.52	15/12	

Neuronal activity-associated Ca^{2+} influx in burst and regular spiking subicular neurons of WT and 3xTG mice. n indicates the number of recorded neurons, N represents the number of recorded mice. Statistical differences according to Mann-Whitney test.

Table S11. Low-voltage activated Ca^{2+} currents in subicular neurons (refers to Figure S5).

Low-voltage activated Ca^{2+} currents in subicular neurons

		before 4 μM Z944			after 4 μM Z944				
		Median	Minimum	Maximum	Median	Minimum	Maximum	n/N	Wilcoxon test
WT	current density (pA/pF)	2.56	1.39	5.84	0.04	0	0.09	6/3	*0.0313
3xTG	current density (pA/pF)	2.19	0.81	6.13	0.06	0	0.12	7/3	**0.0078

Burst frequency of subicular neurons

		before 4 μM Z944			after 4 μM Z944				
		Median	Minimum	Maximum	Median	Minimum	Maximum	n/N	Wilcoxon test
WT	intra-burst frequency (Hz)	202.8	125.5	294.4	0	0	0	14/8	****0.0001
3xTG	intra-burst frequency (Hz)	200.4	151.3	222.2	0	0	0	10/5	***0.0001

Inactivation of low-voltage activated Ca^{2+} currents in subicular neurons

		Median	Minimum	Maximum	n/N	Mann-Whitney test
WT	current density (pA/pF)	2.1	0.33	8.17	17/7	0.0851
3xTG	current density (pA/pF)	1.5	0.18	4.42	17/6	
WT	tau (ms)	44.95	23.94	127.7	17/7	*0.0344
3xTG	tau (ms)	73.67	31.94	142.8	17/6	

Steady-state voltage-dependency of low-voltage activated Ca^{2+} currents in subicular neurons

		Median	Minimum	Maximum	n/N	Mann-Whitney test
WT	V50 activation (mV)	-64.29	-73.19	-58.01	17/7	*0.0344
3xTG	V50 activation (mV)	-60.23	-73.02	-57.43	17/6	
WT	slope of activation	3.99	0.76	8.65	17/7	0.1932
3xTG	slope of activation	2.39	0.91	9.3	17/6	
WT	V50 inactivation (mV)	-90.64	-102.2	-74.53	17/7	0.9999
3xTG	V50 inactivation (mV)	-91.62	-109.9	-69.23	17/6	
WT	slope of inactivation	6.71	2.75	10.16	17/7	0.6584
3xTG	slope of inactivation	6.54	3.66	10.12	17/6	

Pharmacological and biophysical characterization of low-voltage activated Ca^{2+} currents in subicular neurons of WT and 3xTG mice. n indicates the number of recorded neurons, N represents the number of recorded mice. Statistical differences according to Mann-Whitney test.

Table S12. Neuronal activity-associated T-type channels mediated Ca^{2+} influx in subicular neurons (refers to Figure S6).

Neuronal activity-associated somatic Ca^{2+} influx

			before 4 μM Z944			after 4 μM Z944				
			Median	Minimum	Maximum	Median	Minimum	Maximum	n/N	Wilcoxon test
WT	UCP4 -	ΔG/R	0.27	0.08	0.54	0.17	0.06	0.4	10/4	**0.002
	UCP4 +	ΔG/R	0.39	0.18	0.72	0.22	0.15	0.69	9/4	*0.0273
3xTG	UCP4 -	ΔG/R	0.28	0.17	0.52	0.26	0.14	0.4	9/7	**0.0078
	UCP4 +	ΔG/R	0.24	0.1	0.76	0.25	0.17	0.52	5/4	0.8125

Neuronal activity-associated dendritic Ca^{2+} influx

			before 4 μM Z944			after 4 μM Z944				
			Median	Minimum	Maximum	Median	Minimum	Maximum	n/N	Wilcoxon test
WT	UCP4 -	ΔG/R	0.15	0.08	0.32	0.14	0.04	0.3	7/4	0.2969
	UCP4 +	ΔG/R	0.25	0.03	0.76	0.54	0.03	1.06	7/4	*0.0313
3xTG	UCP4 -	ΔG/R	0.17	0.11	0.67	0.31	0.03	0.83	9/7	>0.9999
	UCP4 +	ΔG/R	0.19	0.06	1.12	0.15	0.07	0.48	5/4	0.3125

			Z944-sensitive somatic current				Z944-sensitive dendritic current			
			Median	Minimum	Maximum	n/N	Median	Minimum	Maximum	n/N
WT	UCP4 -	%	27.42	8.7	60.31	10/4	18.57	-88.52	55.8	7/4
	UCP4 +	%	15.84	-0.62	39.04	9/4	-26	-165.4	0.22	7/4
3xTG	UCP4 -	%	6.6	-19.87	36.58	9/7	13.74	-174.8	67.87	9/7
	UCP4 +	%	10.01	-4.37	32.37	5/4	34.73	-27.7	56.48	5/4

2-way ANOVA
Z944-sensitive somatic current

	F (DFn, DFd)	p value
Interaction	F (1, 27) = 2.344	0.1374
Genotype	F (1, 27) = 1.742	0.198
Treatment	F (1, 27) = 2.989	0.0952

2-way ANOVA
Z944-sensitive dendritic current

	F (DFn, DFd)	p value
Interaction	F (1, 24) = 5.328	*0.0299
Genotype	F (1, 24) = 1.945	0.1759
Treatment	F (1, 24) = 1.041	0.3177

Uncorrected Fisher's LSD test<br>Z944-sensitive somatic current			
WT UCP4± vs 3xTG UCP4±			p value
WT UCP4 -	vs	WT UCP4 +	*0.0205
WT UCP4 -	vs	3xTG UCP4 -	*0.0288
WT UCP4 -	vs	3xTG UCP4 +	*0.048
WT UCP4 +	vs	3xTG UCP4 -	0.7648
WT UCP4 +	vs	3xTG UCP4 +	0.8941
3xTG UCP4 -	vs	3xTG UCP4 +	0.8959

Uncorrected Fisher's LSD tests<br>Z944-sensitive dendritic current			
WT UCP4± vs 3xTG UCP4±			p value
WT UCP4 -	vs	WT UCP4 +	*0.0242
WT UCP4 -	vs	3xTG UCP4 -	0.4905
WT UCP4 -	vs	3xTG UCP4 +	0.8072
WT UCP4 +	vs	3xTG UCP4 -	0.0765
WT UCP4 +	vs	3xTG UCP4 +	*0.0223
3xTG UCP4 -	vs	3xTG UCP4 +	0.3813

Neuronal activity-associated somatic Ca^{2+} influx

		before 5 mM caffeine			after 5 mM caffeine				
		Median	Minimum	Maximum	Median	Minimum	Maximum	n/N	Wilcoxon test
WT	ΔG/R	0.32	0.12	0.49	0.5	0.14	0.68	10/8	**0.002
3xTG	ΔG/R	0.16	0.07	0.45	0.26	0.11	0.55	8/6	**0.0078

		Caffeine-evoked somatic current				Mann-Whitney test
		Median	Minimum	Maximum	n/N	
WT	%	26.13	5.87	52.08	10/8	0.9999
3xTG	%	28.14	14.22	56.42	8/6	

Neuronal activity-associated dendritic Ca^{2+} influx

		before 5 mM caffeine			after 5 mM caffeine				
		Median	Minimum	Maximum	Median	Minimum	Maximum	n/N	Wilcoxon test
WT	ΔG/R	0.14	0.07	0.52	0.17	0.05	1.14	10/8	0.1934
3xTG	ΔG/R	0.11	0.08	0.33	0.33	0.09	0.64	8/6	0.0781

		Caffeine-evoked dendritic current				Mann-Whitney test
		Median	Minimum	Maximum	n/N	
WT	%	22.78	-96.6	62.57	10/8	0.3599
3xTG	%	39.31	-18.95	80.99	8/6	

Somatic and dendritic neuronal activity-associated Ca^{2+} influx through T-type Ca^{2+} channels in subicular neurons of WT, WT overexpressing UCP4, 3xTG and 3xTG overexpressing UCP4 mice. n indicates the number of recorded neurons, N represents the number of recorded mice. Statistical differences according to Wilcoxon test and 2-way ANOVA followed by Fishers LSD multiple comparisons test.

Somatic and dendritic neuronal activity-associated Ca^{2+} influx before and after caffeine application in subicular neurons of WT and 3xTG mice. n indicates the number of recorded neurons, N represents the number of recorded mice. Statistical differences according to Wilcoxon and Mann-Whitney test.

Table S13. Changes in neuronal firing during long-term whole-cell recordings under control conditions (refers to Figure S7).

Neuronal firing frequency in long-term whole-cell recordings

		ACSF (t10) (=before 2 nM GDNF)			ACSF (t50) (=after 2 nM GDNF)				
		Median	Minimum	Maximum	Median	Minimum	Maximum	n/N	Wilcoxon test
WT	Firing frequency (Hz)	22.92	10.42	34.38	20.83	10.42	48.96	11/4	0.168
3xTG	Firing frequency (Hz)	21.88	3.12	36.46	20.83	3.12	40.63	9/4	0.6406

Neuronal firing frequency before and after 40min washing in ACSF in WT and 3xTG mice. n indicates the number of recorded neurons, N represents the number of recorded mice. Statistical differences according to Wilcoxon test.
